# Analysis of growth dynamics in five different media and metabolic phenotypic characteristics of *Piriformospora indica*

**DOI:** 10.3389/fmicb.2023.1301743

**Published:** 2024-01-08

**Authors:** Jing-rong Hu, Jin-meng Li, Hai-yan Wang, Mei-li Sun, Chun-yang Huang, Han-cheng Wang

**Affiliations:** ^1^Institute of Advanced Agricultural Science, Hubei University of Arts and Science, Xiangyang, Hubei, China; ^2^College of Agriculture, Yangtze University, Jingzhou, China; ^3^Guizhou Provincial Tobacco Company, Zunyi Branch, Zunyi, China; ^4^Guizhou Provincial Academician Workstation of Microbiology and Health, Guizhou Academy of Tobacco Science, Guiyang, Guizhou, China

**Keywords:** *Piriformospora indica*, growth and sporulation, Biolog Phenotype Microarray, metabolic profiling, culture media

## Abstract

*Piriformospora indica* is an important endophytic fungus with broad potential for alleviating biotic and abiotic stress on host plants. This study monitored the growth dynamics of *P. indica* on five commonly used artificial media for microorganisms and analyzed its metabolic characteristics using Biolog Phenotype Microarray (PM) technology. The results showed that *P. indica* grew fastest on Potato Dextrose Agar (PDA), followed by Kidney Bean Agar (KBA), Alkyl Ester Agar (AEA), Oatmeal Agar (OA), and Luria-Bertani Agar (LB), and the most suitable medium for spore production was OA. Using Biolog PM1-10, 950 metabolic phenotypes of *P. indica* were obtained. *P. indica* could metabolize 87.89% of the tested carbon sources, 87.63% of the tested nitrogen sources, 96.61% of the tested phosphorus sources, and 100% of the tested sulfur sources. *P. indica* displayed 92 kinds of tested biosynthetic pathways, and it could grow under 92 kinds of tested osmotic pressures and 88 kinds of tested pH conditions. PM plates 1-2 revealed 43 efficient carbon sources, including *M*-Hydroxyphenyl acid, *N*-Acetyl-*D*-Glucosamine, Tyramine, Maltotrios, α*-D*-Glucosine, *I*-Erythritol, *L*-Valine, *D*-Melezitose, *D*-Tagatose, and Turanose. PM plates 3,6-8 indicated 170 efficient nitrogen sources, including Adenosine, Inosine Allantoin, *D, L*-Lactamide, Arg-Met, lle-Trp, Ala-Arg, Thr-Arg, Trp-Tyr, Val-Asn, Gly-Gly-*D*-Leu, Gly-Gly-Phe, and Leu-Leu-Leu. This study demonstrates that *P. indica* can metabolize a variety of substrates, such as carbon and nitrogen sources, and has a wide range of environmental adaptability. The growth dynamics on artificial culture media and metabolic phenotypes of *P. indica* can be used to investigate its biological characteristics, screen for more suitable growth and sporulation conditions, and elucidate the physiological mechanisms that enhance the stress resistance of host plants. This study provides a theoretical basis for its better application in agriculture.

## Introduction

*Piriformospora indica* belongs to the genus *Piriformospora*, the family *Sebacinaceae*, the order *Sebacinales*, the class *Hymenomycetes*, and the phylum *Basidiomycota* (Waller et al., 2005; Hibbett et al., 2007). It is a root endophytic fungus that was isolated by Indian scientists from the rhizosphere of woody shrubs while collecting spores of the arbuscular mycorrhiza (AM) fungus *Glomus mosseae* in the interior desert of Rajasthan, India (Verma et al., 1998). It is named *P. indica* due to its pear-shaped chlamydospores (Varma et al., 2012). It shares similarities with some AM fungi, such as *G. mosseae* and *Gigaspora margarita* (Chen et al., 2007; Venice et al., 2020). They are both endophytic fungi that can coexist with the host plant (Singh et al., 2000; Kesharwani et al., 2022), promote the absorption of mineral elements and cycling substances in the soil, and enhance the plant's resistance to biotic and abiotic stress (Xu et al., 2018; Bandyopadhyay et al., 2022). Compared to the AM fungus, *P. indica* has notable advantages, such as its ability to obtain nutrition from non-living sources and its wide range of host plants (Varma et al., 2001; Deshmukh et al., 2006; Oelmüller et al., 2009). Therefore, it has gained widespread attention since its discovery (Kaldorf et al., 2005; Gill et al., 2016; Su et al., 2017). *P. indica* can interact with various plant species, including *Gymnospermae* and *Angiospermae* (Khalid et al., 2019), as well as lower plants such as *Bryophyta* and *Pteridophyta* (Varma et al., 1999; Manzar et al., 2021a). Currently, it is known to colonize over 200 plants, including monocotyledonous plants such as *Oryza sativa* (Ghorbani et al., 2021), *Triticum aestivum* (Li et al., 2022), *Hordeum vulgare* (Alikhani et al., 2013), and *Zea mays* (Xu et al., 2017), and dicotyledonous plants like *Nicotiana tabacum* (Hui et al., 2015), *Passiflora edulis* (Yan et al., 2021), *Solanum tuberosum* (Fakhro et al., 2010), and *Cicer arietinum* (Narayan et al., 2017). It can even colonize *Arabidopsis thaliana*, a *cruciferous* model plant that AM fungi cannot colonize (Abdelaziz et al., 2017; Manzar et al., 2021b). Many scholars have studied the colonization of *P. indica* in various plants, focusing on its effects on promoting host plant growth, improving disease resistance, and enhancing stress tolerance, including resistance to drought, cold, heat, salt, and flooding stresses (Ghabooli et al., 2013; Amani et al., 2021). However, research on the basic biological characteristics of *P. indica*, such as its growth dynamics on different culture media, has been limited and has only been focused on screening suitable media at a macro level. There have been no reports on the micro-metabolic phenotype profiling of *P. indica* up to now.

The metabolic phenotypes include the ability to utilize carbon, nitrogen, phosphorus, and sulfur sources, as well as growth tests under different osmotic pressure and pH conditions (Mazur et al., 2013; Narware et al., 2023). The Phenotype Microarray (PM) technology, developed by Biolog Company in the United States, can be used to determine various metabolic phenotypes of microorganisms. The PM is a novel technology that is parallel to genomics and proteomics and is widely used in the study of various microorganisms (Bochner, 2003). It involves fixing a dried cell culture medium and a colorimetric substance in each well of a 96-well microplate (PM plate). After adding microbial cell suspension and incubation, the cell metabolic phenotype can be observed through color changes. Finally, the dynamics of phenotypic reactions are obtained, and the strength of phenotypic reactions can be quantitatively measured (Li and Lu, 2007).

Based on these premises, this study aimed at screening the most suitable growth medium and sporulation medium for *P. indica* by analyzing its growth dynamics on five commonly used artificial culture media. The goal is to quickly provide the required biomass and spore quantities for production or scientific research. Additionally, the metabolic phenotype of *P. indica* was assessed using the Biolog PM technology, aiming to identify its characteristic nutrients and reveal its adaptability under different osmotic pressure and pH environments. This information will help to clarify the physiological reasons behind its enhancement of plant stress resistance and provide a theoretical basis for the development and utilization of *P. indica* as a biocontrol agent.

## Materials and methods

### Fungal strain, culture media, and reagents

The tested strain of *Piriformospora indica* was provided by the College of Life Sciences, Yangtze University (Dong et al., 2013). The five test media were formulated as follows: Potato Dextrose Agar (PDA): potato 200 g·L^−1^, glucose 20 g·L^−1^, and agar 20 g·L^−1^. Alkyl Ester Agar (AEA): yeast extract 5 g·L^−1^, glycerol 20 ml·L^−1^, MgSO_4_ 0.25 g·L^−1^, NaNO_3_ 6 g·L^−1^, KCl 0.5 g·L^−1^, KH_2_PO_4_ 1.5 g·L^−1^, and agar 20 g·L^−1^. Oatmeal Agar (OA): raw oatmeal powder 30 g·L^−1^ and 20 g·L^−1^ agar. Kidney Bean Agar (KBA): kidney bean 150 g·L^−1^ and agar 20 g·L^−1^. Luria-Bertani (LB): tryptone 10 g·L^−1^, yeast extract 5 g·L^−1^, and NaCl 10 g·L^−1^. All culture media were sterilized at 116°C for 30 min.

PM1-10 metabolic plates (Grades: # 12111, # 12112, # 12121, # 12131, # 12141, # 12181, # 12182, # 12183, # 12161, and # 12162), sterile sampling tank (# 3002), FF-IF inoculum (# 72106), OmniLog PM system (# 91171), a turbidity meter (# 3531), and an 8-channel electric pipette (# 3501A) were produced by Biolog, Hayward, CA, United States.

### Growth and sporulation monitoring of *P. indica* on five different culture media

The plates of *P. indica* preserved at 4°C were inoculated onto new PDA plates and incubated at 28°C for 1 week. Using sterile punchers (Ø = 6 mm), fungal blocks were made from the active edges of the fungal colonies and then injected into separate Petri dishes (Ø = 12 cm), containing different solid culture media. Fifteen plates were inoculated for each type of culture medium (PDA, AEA, OA, KBA, and LB), and all plates were placed in a dark incubator at 28°C. The diameter of the colony on each plate was measured daily using the vertical cross method; three repeats were set for each medium. The remaining plates were used for photography and microscopic examination to observe spore production and determine the quantity of spores produced. A small amount of mycelium was taken daily with a sterile toothpick, stained with 0.05% trypan blue for 5 min, washed twice with sterile water, and observed for sporulation and the amount of spore production under a 400× microscope (Nikon, Y-TV55, Japan).

### Biolog PM metabolic phenotype detection of *P. indica*

The Biolog PM test involved 950 different conditions, including 190 carbon sources (PM1-2), 380 nitrogen sources (PM3, 6-8), 94 biosynthetic pathways (PM5), 59 phosphorus sources, 35 sulfur sources (PM4), 96 osmotic pressures (PM9), and 96 pH conditions (PM10). The phenotypic analysis of *P. indica* was conducted following the Biolog standard procedures (Bochner, 2003). *P. indica* was incubated on an OA plate under dark conditions at 28°C. After 5 days, spores were produced on the surface of the colony. Sterile cotton swabs moistened with sterile water were used to rotate and dip the spores on the colony's surface, and they were diluted to a 12-ml FF-IF inoculum. The concentration of the spore suspension was adjusted to 62% T (T is the Biolog standard concentration unit) (Bochner, 2003).

### Carbon metabolism phenotype analysis

A total of 0.05 ml of spore suspension was added to 23.95 ml of PM1 and PM2 inoculum, vortexed, and mixed evenly. An 8-channel electric pipette was used to add the mixed spore suspension to PM1 and PM2 metabolic plates, with 100 μl per well. The prepared phenotypic assay plate was incubated for 6 days at 28°C in an OmniLog constant-temperature incubator. The OmniLog software was set up to collect data. The carbon source metabolism phenotype of *P. indica* was analyzed based on its metabolic kinetics curve.

### Nitrogen metabolism and pathway phenotype analysis

A total of 0.125 ml of spore suspension was added to 59.875 ml of PM3, PM5, PM6, PM7, and PM8 inoculum, vortexed, and mixed evenly. The spore suspension was added to the metabolic plates and incubated, and the data were collected as described above. The nitrogen source metabolism and pathway phenotype of *P. indica* were analyzed based on its metabolic kinetics curve.

### Phosphorus and sulfur metabolism phenotype analysis

A total of 0.025 ml of spore suspension was added to 11.975 ml of PM4 inoculum, vortexed, and mixed evenly. The spore suspension was added to the metabolic plates and incubated, and the data were collected as described above. The phosphorus and sulfur metabolism phenotypes of *P. indica* were analyzed based on its metabolic kinetics curve.

### Osmotic pressure and pH metabolism phenotype analysis

A total of 0.05 ml of spore suspension was added to 23.95 ml of PM9 and PM10 inoculum, vortexed, and mixed evenly. The spore suspension was added to the metabolic plates and incubated, and data were collected as described above. The osmotic pressure and pH metabolism phenotype of *P. indica* were analyzed based on its metabolic kinetics curve.

### Data processing and metabolism phenotype analysis

Data analysis was performed using SPSS 17.0, and Excel 2007 was used to simulate the growth curve regression equation. The results were considered significant when means were assessed with Duncan's new multiple-range test at *p* < 0.05. Values are the means ± SD of three replications. The Biolog D5E_OKA_data.exe software was used to collect metabolic phenotype data for *P. indica*, and the Biolog OL_FM_1.2.exe software was used for data conversion. The size of the color area of the metabolic wells was used to analyze the metabolic phenotype characteristics of *P. indica*. Substrate utilization rate (%) = number of available substrate wells/(total number of wells with substrate – number of control) × 100%.

## Results

### Growth dynamics and sporulation results of *P. indica* on five different culture media

**Figure 1:**
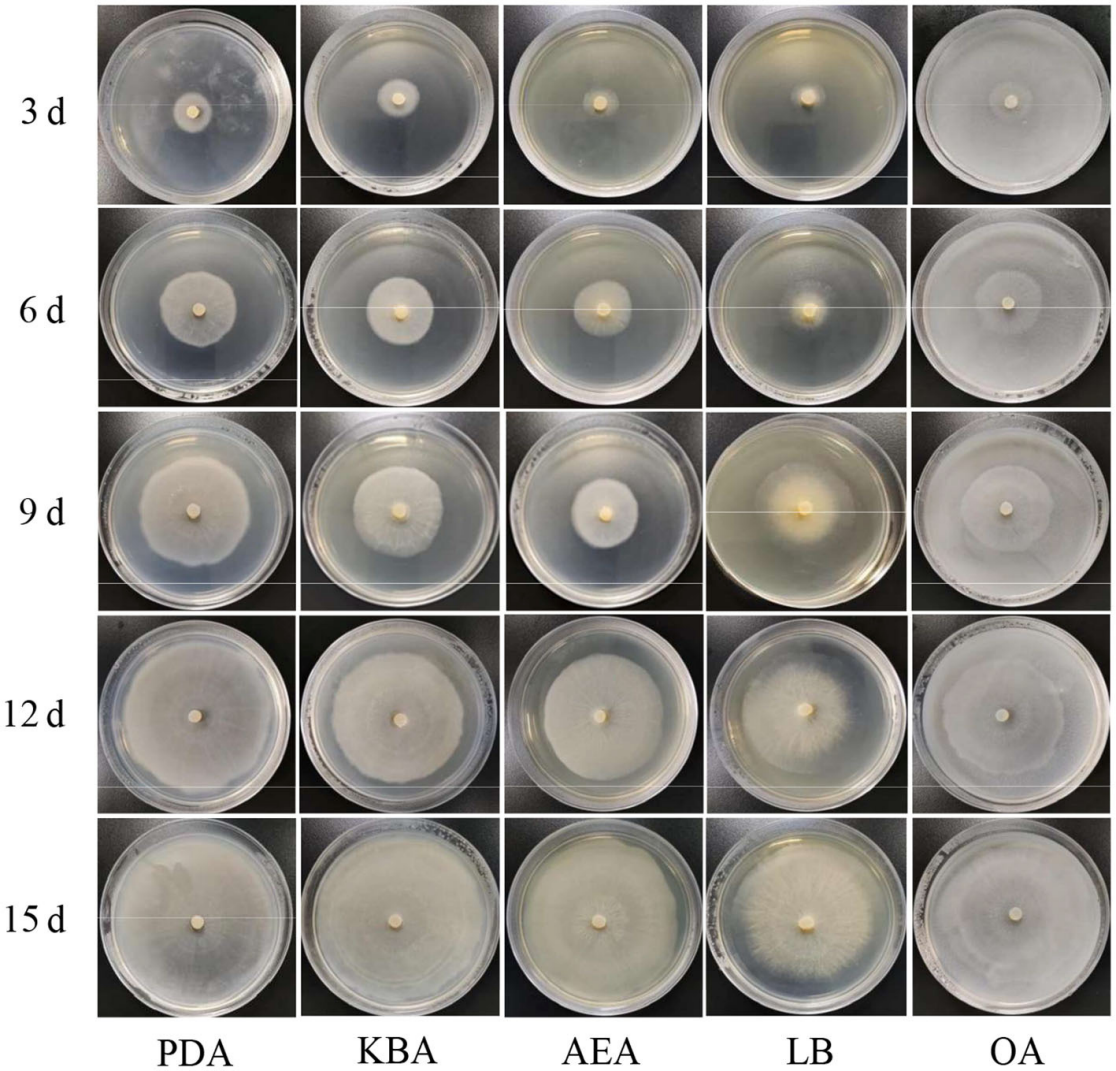
Morphology of *Piriformospora indica* cultured on five different media (PDA, KBA, AEA, LB, and OA) for 3, 6, 9, 12, and 15 days.

**Figure 2:**
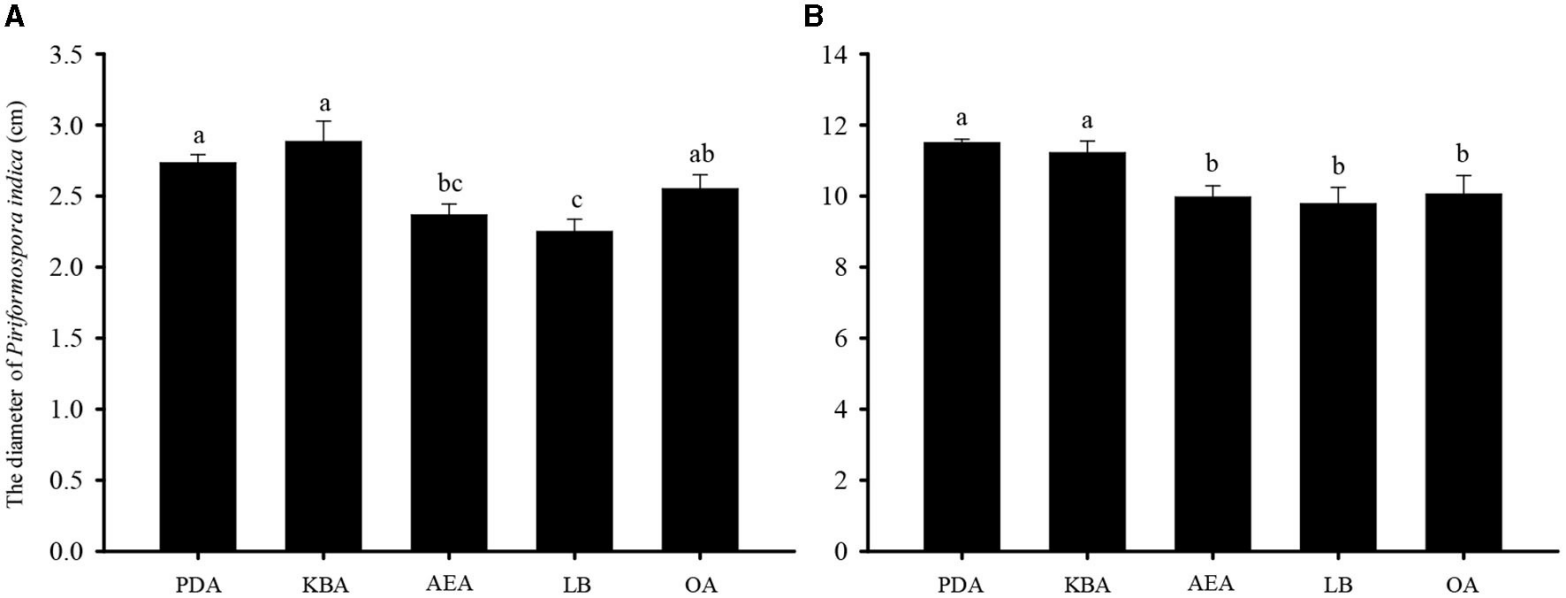
The diameter of *Piriformospora indica* cultured on five different media (PDA, KBA, AEA, LB, and OA) for 3 **(A)** and 15 **(B)** days.

**Figure 3:**
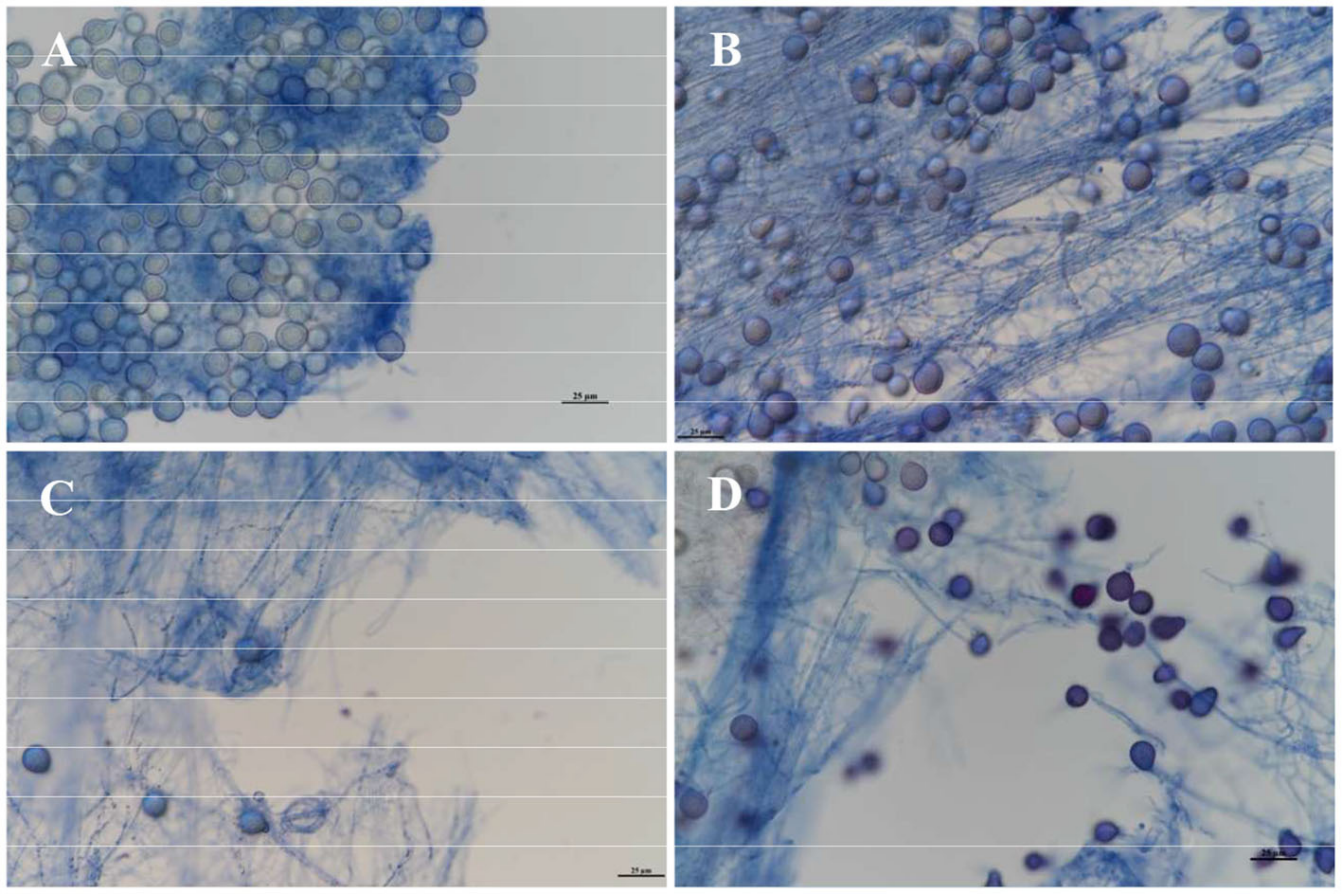
Observation of spore yield of *Piriformospora indica* cultured on different media on the 10th day under a microscope. **(A)** Stands for OA medium, **(B)** stands for KBA medium, **(C)** stands for AEA medium, and **(D)** stands for PDA medium.

The results of the growth dynamics measurement of *P. indica* indicated a linear relationship between colony diameter (cm) and growth time (d) (Table 1). The growth rates in different cultural media were as follows: PDA > KBA > AEA > OA > LB (Figures 1, 2). Among them, it took 14 days, 17 days, 18 days, 19 days, and 20 days for *P. indica* to fully cover the culture dish (Ø = 12 cm) on PDA, KBA, AEA, OA, and LB, respectively. However, spores were first observed on the 4th day on the OA medium, followed by KBA on the 6th day, PDA on the 9th day, and AEA on the 10th day, and no spores were observed on the LB medium. Microscopic observations on the 10th day revealed that OA and KBA media had the highest spore yield (Figure 3). This indicated that among these five culture media, PDA was the most suitable for the growth of *P. indica*, while the OA medium was the most suitable for sporulation, followed by KBA. The OA medium required the shortest time for spore formation, and both the OA and KBA media had the highest spore yield.

**Table 1:** Growth dynamics and spore production of *Piriformospora indica* on five different media.

**Culture media**	**Regression equations**	**R^2^**	**Spore formation time**	**Sporulation quantity on the 10th day**
PDA	y = 0.8661x + 0.3496	0.9886	≤9 days	+ +
KBA	y = 0.7175x + 0.73	0.9927	≤6 days	+ + +
AEA	y = 0.6018x + 0.5927	0.9918	≤10 days	+
OA	y = 0.5969x + 0.9368	0.9843	≤4 days	+ + +
LB	y = 0.5936x + 0.7952	0.9752	-	−

Sporulation quantity: + + + represents sporulation is abundant, + + represents sporulation is moderate, + represents sporulation is low, − represents no sporulation.

### Metabolic phenotypic features of *Piriformospora indica*

**Figure 4:**
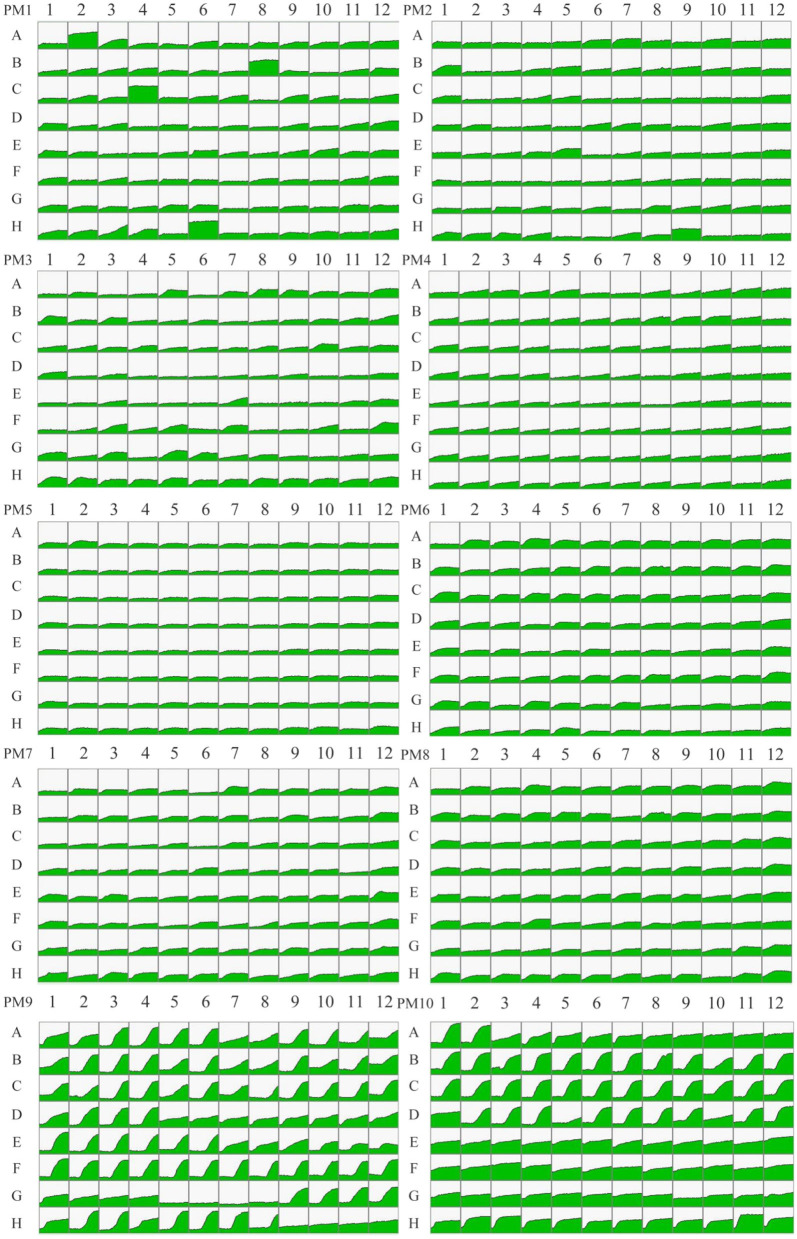
The metabolic fingerprint of microarray PM1-10 for *Piriformospora indica*. PM1-2 stands for carbon metabolic phenotype, PM3 and PM6-8 stands for nitrogen metabolic phenotype, PM4 Wells A01-E12 stands for phosphorous metabolic phenotype, PM4 Wells F01-H12 stands for sulfur metabolic phenotype, PM5 stands for biosynthetic pathway phenotype, PM9 stands for osmotic pressure phenotype, and PM10 stands for pH phenotype. Green areas represent the metabolic fingerprint of *P. indica* in each substrate; the larger the green proportion, the higher the efficiency of utilization.

Fingerprinting patterns and metabolic data for *P. indica* were obtained, resulting in 950 pieces of information on metabolic phenotypes. Kinetic response curves reflecting microbial growth were generated for each well in PM1-10. Green areas represent the metabolic fingerprint of *P. indica* in each substrate; the larger the green proportion, the higher the efficiency of utilization (Figure 4). The results indicated that *P. indica* could utilize 87.89% of the tested carbon sources (76/95 in plate PM1 and 91/95 in plate PM2), 87.63% of the tested nitrogen sources (93/95 in plate PM3, 95/95 in plate PM6, 92/95 in plate PM7, and 53/95 in plate PM8), 96.61% of the tested phosphorus sources (57/59 in plate PM4), and 100% of the tested sulfur sources (35/35 in plate PM4), with 92 biosynthetic pathways tested. It was able to grow under 92 tested osmotic pressures and 88 tested pH values, demonstrating a wide range of substrate metabolism capability and strong environmental adaptability.

### Metabolic phenotypic analysis of *Piriformospora indica* on carbon sources

A total of 190 pieces of metabolic phenotypic information about carbon sources were obtained through metabolomics analysis. *P. indica* could metabolize 167 carbon sources, and 43 carbon sources were efficiently utilized (5/95 in plate PM1 and 38/95 in plate PM2; Table 2), including *M*-Hydroxyphenyl acetic acid, *N*-Acetyl-*D*-Glucosamine, Tyramine, Maltotrios, α*-D*-Glucose, *N-*Acetyl*-D-*Galactosamine, *L*-Valine, *D*-Melezitose, *D*-Tagatose, and Turanose. There were 75 moderately efficient carbon sources (39/95 in plate PM1 and 36/95 in plate PM2). The sporulation rate of *P. indica* on highly efficient carbon sources was 79.07% (4/5 in plate PM1 and 30/38 in plate PM2), and the overall sporulation rate among all metabolizable carbon sources was 70.66% (58/76 in plate PM1 and 60/91 in plate PM2). Only 23 carbon sources could not be metabolized by *P. indica* in all tested carbon substrates, including Methyl pyruvate, *D*-Glucose-1-Phosphate, *L*-Lyxose, *D*-Psicose, α-Methyl-*D*-Galactoside, *D*-Glucuronic acid, *D*-Lactic acid methyl ester, and 2-Hydroxybenzoic acid.

**Table 2:** Carbon substrates efficiently metabolized by *Piriformospora indica* and sporulation results in PM1-2.

**Carbon substrate**	**Sporulation**	**Well**	**Carbon substrate**	**Sporulation**	**Well**
**PM1**					
*M*-Hydroxyphenylacetic acid	+	H03	Maltotriose	−	E10
*N-*Acetyl*-D-*Glucosamine	+	A03	α-D-Glucose	+	C09
Tyramine	+	H04			
**PM2**					
*N-*Acetyl*-D-*Galactosamine	−	B01	*L*-Isoleucine	+	G09
*L*-Valine	+	H04	Dextrin	+	A06
*D*-Melezitose	+	C04	*L*-Histidine	+	G06
*D*-Tagatose	+	D06	*L*-Lysine	−	G11
Turanose	+	D07	Arbutin	−	B08
*L*- Phenylalanine	+	H02	*I*-Erythritol	+	B10
*L*-Arabito	+	B07	Citraconic acid	+	E03
*L-*Octopamine	+	H07	*N-*Acetyl*-L-*Glutamic acid	+	G03
Gelatin	−	A07	Gentiobiose	+	C01
γ-Aminobutyric acid	+	D10	Salicin	+	D02
*D*-Glucosamine	+	E05	Citramalic acid	−	E04
*L*-Histidine	+	G08	Sebacic acid	+	F08
*L*-Leucine	+	G10	*L*-Pyroglutamic acid	+	H03
*L*-Ornithine	−	H01	*D*-Arabitol	+	B06
Laminarin	+	A10	*D*-Arabinose	+	B05
Amygdalin	−	B04	Quinic acid	+	F06
Maltitol	+	C05	Succinamic acid	−	F10
4-Hydroxybenzoic acid	+	E07	*D*-Tartaric acid	+	F11
*L*-Arginine	+	G04	β-Hydroxy butyric acid	+	E08

+ represents spores are produced, − represents no sporulation.

### Metabolic phenotypic analysis of *Piriformospora indica* on nitrogen sources

A total of 380 pieces of nitrogen metabolic phenotypic information (PM3 and PM6-8) were obtained through metabolomics analysis. *P. indica* was able to metabolize 333 nitrogen sources, with 170 kinds of them being efficiently utilized (38/95 in plate PM3, 63/95 in plate PM6, 54/95 in plate PM7, and 15/95 in plate PM8; Table 3). This included substances such as Adenosine, Inosine Allantoin, *D, L*-Lactamide, Arg-Met, lle-Trp, Ala-Arg, Thr-Arg, Trp-Tyr, Val-Asn, Gly-Gly-D-Leu, Gly-Gly-Phe, and Leu-Leu-Leu. Interestingly, only Adenosine and *D, L*-Lactamide were found to produce spores among the highly efficient nitrogen sources. A total of 121 nitrogen sources were moderately utilized (36/95 in plate PM3, 30/94 in plate PM6, 33/94 in plate PM7, and 22/94 in plate PM8), and 39 nitrogen sources were weakly utilized (19/95 in plate PM3, 1/94 in plate PM6, 4/94 in plate PM7, and 15/94 in plate PM8). Only 47 nitrogen sources (12.37%) could not be metabolized by *P. indica*, including *N*-Acetyl-*D*-Mannosamine, Guanine, Lys-Pro, Met-Pro, Lys-Gly, Pro-Ile, *D*-Ala-Leu, Val-Pro, *D*-Leu-Gly, and *D*-Ala-Gly-Gly.

**Table 3:** Nitrogen substrates efficiently metabolized by *Piriformospora indica* and sporulation results in PM3, 6-8.

**Nitrogen substrate**	**Sporulation**	**Well**	**Nitrogen substrate**	**Sporulation**	**Well**
**PM3**					
Adenosine	+	F03	Ala-Glu	−	H03
Inosine	−	F12	Gly-Glu	−	H10
Allantoin	−	G05	*N*-Acetyl-*D, L*-Glutamic acid	−	D01
*D, L*-Lactamide	+	E07	Met-Ala	−	H12
*L*-Tryptophan	−	B12	*L*-Glutamic acid	−	A12
Cytosine	−	F05	Ala-Thr	−	H07
Ala-His	−	H05	Gly-Gln	−	H09
*L*-Arginine	−	A08	*L*-Valine	−	C02
Uracil	−	F10	*L*-Asparagine	−	A09
Ala-Asp	−	H01	*L*-Threonine	−	B11
Ala-Gln	−	H02	*D*-Asparagine	−	C04
Urea	−	A05	Ala-Gly	−	H04
Gly-Asn	−	H08	Cytidine	−	F04
*L*-Histidine	−	B03	Gly-Met	−	H11
*L*-Glutamine	−	B01	Tyramine	−	E03
Uric acid	−	G03	Adenine	−	F02
*L*-Citrulline	−	C10	*D, L-α-*Amino*-N-*Butyric acid	−	G07
Ala-Leu	−	H06	*L*-Aspartic acid	−	A10
*D*-Aspartic acid	−	G06	*L*-Pyroglutamic acid	−	D03
**PM6**					
Arg-Met	−	C01	Gly-Arg	−	E03
lle-Trp	−	H01	Ala-Phe	−	A11
Ala-Arg	−	A04	Ala-Trp	−	B03
Arg-Asp	−	B07	Ala-Tyr	−	B04
Arg-Lys	−	B12	Arg-Phe	−	C02
Asp-Phe	−	D01	Asp-Trp	−	D02
Glu-Tyr	−	D12	Ile-Gln	−	G05
His-Lys	−	F08	Gln-Gly	−	D06
His-Trp	−	F12	Gly-Thr	−	F01
Arg-Gln	−	B08	Arg-Ile	−	B10
Arg-Arg	−	B06	Asp-Lys	−	C12
Arg-Ala	−	B05	Glu-Trp	−	D11
His-Tyr	−	G01	Gly-Trp	−	F02
Arg-Trp	−	C04	Ala-Gly	−	A07
Arg-Tyr	−	C05	Ile-Tyr	−	H02
His-Leu	−	F07	Ala-His	−	A08
Ile-Arg	−	G04	Arg-Leu	−	B11
Leu-Arg	−	H05	Asn-Glu	−	C07
Arg-Ser	−	C03	His-Ser	−	F11
Asp-Val	−	D03	Leu-Ala	−	H04
Glu-Val	−	E01	Leu-Phe	−	H12
His-Met	−	F09	Glu-Glu	−	D08
His-Asp	−	F05	Ala-Ser	−	B01
His-Gly	−	F06	Gly-Ser	−	E12
His-Val	−	G02	Ile-Val	−	H03
Ile-His	−	G07	Ala-Ala	−	A03
Gln-Gln	−	D05	Asp-Asp	−	C09
Gly-Val	−	F04	Asp-Glu	−	C10
Ala-Asn	−	A05	Gly-His	−	E06
Arg-Glu	−	B09	Gly-Tyr	−	F03
Arg-Val	−	C06	His-Pro	−	F10
Asn-Val	−	C08			
**PM7**					
Thr-Arg	−	E12	Met-Pro	−	C08
Trp-Tyr	−	G04	Trp-Ala	−	F06
Val-Asn	−	H04	Trp-Leu	−	F11
Trp-Asp	−	F08	Trp-Phe	−	G01
Pro-Gln	−	D06	Val-Gly	−	H05
Tyr-Trp	−	H01	Lys-Leu	−	A10
Val-Arg	−	H03	Met-Gln	−	B09
Val-Gly	−	H06	Tyr-Glu	−	G07
Lys-Arg	−	A07	Tyr-Gly	−	G08
Met-His	−	B12	Val-Val	−	H11
Val-Leu	−	H09	Pro-Leu	−	D09
Trp-Lys	−	F12	Ser-Pro	−	E07
Val-His	−	H07	Ser-Tyr	−	E09
Tyr-Gln	−	G06	Trp-Glu	−	F09
Pro-Tyr	−	D12	Lys-Ile	−	A09
Tyr-Phe	−	G12	Lys-Trp	−	B04
Ser-His	−	E03	Pro-Phe	−	D10
Tyr-His	−	G09	Tyr-Lys	−	G11
Tyr-Ala	−	G05	Tyr-Tyr	−	H02
Val-Tyr	−	H10	Lys-Glu	−	A08
Tyr-Leu	−	G10	Lys-Thr	−	B03
Met-Trp	−	C07	Phe-Trp	−	D03
Ser-Phe	−	E06	Ser-Met	−	E05
Leu-Trp	−	A04	Lys-Phe	−	A12
Met-Arg	−	B07	Lys-Val	−	B06
Ser-Val	−	E10	Met-Phe	−	C05
*Y*-Glu-Gly	−	H12	Pro-Gly	−	D07
**PM8**					
Gly-Gly-*D*-Leu	−	H02	Gly-Gly-Gly	−	H03
Gly-Gly-Phe	−	H06	Gly-*D*-Thr	−	G06
Leu-Leu-Leu	−	H10	Gly-Gly-Ala	−	H01
Gly-Gly-Ile	−	H04	Val-Tyr-Val	−	H07
Leu-Gly-Gly	−	H09	Gly-*D*-Ala	−	G03
γ-Glu-Gly	−	G01	Phe-β-Ala	−	G10
Gly-*D*-Ser	−	G05	Tyr-Gly-Gly	−	H12
Gly-Gly-Leu	−	H05			

+ represents spores are produced, − represents no sporulation.

### Metabolic phenotypic analysis of *Piriformospora indica* on phosphorus and sulfur sources

A total of 59 phosphorus sources and 35 sulfur sources metabolic phenotypic information were obtained through metabolomics analysis. *P. indica* could metabolize 57 phosphorus sources, with 42 efficiently utilized phosphorus sources (42/59 in plate PM4, Wells A02-E12; Table 4), including *D*-Mannose-1 phosphate, Adenosine-2′,3′-cyclic monophosphate, triple phosphate, phosphate, and trimetaphosphate. There were 12 moderately utilized phosphorus sources (12/59 in plate PM4) and 3 weakly utilized phosphorus sources (3/59 in plate PM4). Only two phosphorus sources (3.39%) could not be metabolized by *P. indica*, namely Thymidine 3′,5′-Cyclic monophosphate and Methylene diphosphonic acid. *P. indica* could metabolize 100% of the sulfur sources, with 34 efficiently utilized sulfur sources (34/35 in plate PM4, Wells F01-H12; Table 4), including Thiourea, *N*-Acetyl-*L*-Cysteine, 1-Thio-β-*D*-Glucose, Taurine, and *p*-Aminobenzene sulfonic acid. There was one moderately utilized sulfur source (1/35 in plate PM4, Wells F01-H12).

**Table 4:** Phosphorus and sulfur substrates efficiently metabolized by *Piriformospora indica* and sporulation results in PM4.

**Phosphorus substrate**	**Sporulation**	**Well**	**Sulfur substrate**	**Sporulation**	**Well**
**PM4**					
Phosphate	−	A02	Sulfate	−	F02
Trimetaphosphate	−	A04	Thiosulfate	−	F03
Tripolyphosphate	−	A05	Tetrathionate	−	F04
Adenosine-2′-monophosphate	−	A08	Thiophosphate	−	F05
Adenosine-3′-monophosphate	−	A09	Dithiophosphate	−	F06
Adenosine-5′-monophosphate	−	A10	*L*-Cysteine	−	F07
Adenosine-2′,3′-cyclic monophosphate	−	A11	*D*-Cysteine	−	F08
Adenosine-3′,5′-cyclic monophosphate	−	A12	*L*-Cysteinyl-glycine	−	F09
Thiophosphate	−	B01	*L*-Cysteic acid	−	F10
Dithiophosphate	−	B02	Cysteamine	−	F11
*D, L*-α-Glycerol phosphate	−	B03	*L*-Cysteine sulfinic acid	−	F12
β-Glycerol phosphate	−	B04	*N*-Acetyl-*L*-Cysteine	−	G01
Carbamyl phosphate	−	B05	*S*-Methyl-*L*-Cysteine	−	G02
*D*-2-Phospho-glyceric acid	−	B06	Cystathionine	−	G03
*D*-3-Phospho-glyceric acid	−	B07	Lanthionine	−	G04
Guanosine-2′-monophosphate	−	B08	Glutathione	−	G05
Guanosine-3′-monophosphate	−	B09	*D, L*-Ethionine	−	G06
Guanosine-5′-monophosphate	−	B10	*L*-Methionine	−	G07
Guanosine-2′,3′-cyclic monophosphate	−	B11	*D*-Methionine	−	G08
Phosphoenol pyruvate	−	C01	Glycyl-*L*-Methionine	−	G09
Phospho-glycolic acid	−	C02	*N*-Acetyl-*D, L*-Methionine	−	G10
*D*-Glucose-1-phosphate	−	C03	*L*-Methionine sulfoxide	−	G11
*D*-Glucose-6-phosphate	−	C04	*L*-Methionine sulfone	−	G12
*D*-Glucosamine-6-phosphate	−	C06	*L*-Djenkolic acid	−	H01
6-Phospho-gluconic acid	−	C07	Thiourea	−	H02
Cytidine-2′-monophosphat	−	C08	1-Thio-β-*D*-Glucose	−	H03
Cytidine-5′-monophosphate	−	C10	*D, L*-Lipoamide	−	H04
Cytidine-2′,3′-cyclic monophosphate	−	C11	Taurocholic acid	−	H05
*D*-Mannose-1-phosphate	−	D01	Taurine	−	H06
*D*-Mannose-6-phosphate	−	D02	Hypotaurine	−	H07
Cysteamine-*S*-phosphate	−	D03	*p*-Aminobenzene sulfonic acid	−	H08
Phospho-*L*-Arginine	−	D04	Butane sulfonic acid	−	H09
*O*-Phospho-*L*-Serine	−	D06	2-Hydroxyethane sulfonic acid	−	H10
*O*-Phospho-*L*-Threonine	−	D07	Tetramethylene sulfone	−	H12
Uridine-3′-monophosphate	−	D09			
Uridine-5′-monophosphate	−	D10			
*O*-Phospho-*D*-Tyrosine	−	E01			
*O*-Phospho-*L*-Tyrosine	−	E02			
Phosphocreatine	−	E03			
*O*-Phosphoryl-Ethanolamin	−	E05			
Thymidine-3′-monophosphate	−	E09			
Thymidine-5′-monophosphate	−	E10			

− represents no sporulation.

### Metabolic phenotypic analysis of the biosynthetic pathways of *Piriformospora indica*

The experimental results of *P. indica* in PM5 showed that it had 92 kinds of tested biosynthetic pathways (92/94 tested, plate PM5, Wells A03-H12). Typical substrates for biosynthetic pathways included Tween 40, *D, L*-Mevalonic acid, Tween 20, Orotic acid, Butyric acid, α-Ketobutyric acid, *D, L*-Carnitine, *D, L-*α-Lipoic acid (oxidized form), and Caprylic acid.

### Metabolic phenotypic analysis of *Piriformospora indica* at different osmotic pressures

A total of 96 pieces of information on osmotic pressure metabolic phenotypes were obtained. *P. indica* was able to grow in 92 osmotic pressure environments (92/96 tested, plate PM9, Wells A01-H12; Table 5), including 1%−10% NaCl, 3%−6% potassium chloride, 2%−5% sodium sulfate, 5%−20% ethylene glycol, 1%−6% sodium formate, 2%−7% urea, 1%−12% sodium lactate, 20–200 mM sodium phosphate (pH 7), 10–100 mM ammonium sulfate (pH 8), 10–100 mM sodium nitrate, and 10–100 mM sodium nitrite. Under the synergistic effect of 6% NaCl, *P. indica* could still grow under various other osmotic substances (plate PM9, Wells B01-C12). It could not grow under the condition of 20–200 mM sodium benzoate (pH 5.2). Spore production circumstances accounted for 82.61% of all osmotic pressure conditions under which growth was normal.

**Table 5:** Metabolic characteristics of *Piriformospora indica* at different osmotic pressures in PM9.

**Substrate**	**Growth**	**Sporulation**	**Well**	**Substrate**	**Growth**	**Sporulation**	**Well**
**PM9**							
NaCl 1%	+++	+	A01	Sodium formate 1%	+++	+++	E01
NaCl 2%	+++	+	A02	Sodium formate 2%	+++	+++	E02
NaCl 3%	+++	+++	A03	Sodium formate 3%	+++	+++	E03
NaCl 4%	+++	+++	A04	Sodium formate 4%	+++	+++	E04
NaCl 5%	+++	+++	A05	Sodium formate 5%	+++	+++	E05
NaCl 5.5%	+++	+++	A06	Sodium formate 6%	+++	+++	E06
NaCl 6%	++	++	A07	Urea 2%	+++	−	E07
NaCl 6.5%	++	++	A08	Urea 3%	++	−	E08
NaCl 7%	+++	+++	A09	Urea 4%	+++	−	E09
NaCl 8%	+++	+++	A10	Urea 5%	++	−	E10
NaCl 9%	+++	+++	A11	Urea 6%	++	−	E11
NaCl 10%	++	++	A12	Urea 7%	+	−	E12
NaCl 6%	+++	+++	B01	Sodium lactate 1%	+++	+++	F01
NaCl 6% +Betaine	+++	+++	B02	Sodium lactate 2%	+++	+++	F02
NaCl 6% +*N-N* Dimethylglycine	+++	+++	B03	Sodium lactate 3%	+++	+++	F03
NaCl 6% + Sarcosine	+++	+++	B04	Sodium lactate 4%	+++	+++	F04
NaCl 6% + Dimethyl Sulfonyl Propionate	+++	+++	B05	Sodium lactate 5%	+++	+++	F05
NaCl 6% + MOPS	+++	+++	B06	Sodium lactate 6%	+++	++	F06
NaCl 6% + Ectoine	+++	+++	B07	Sodium lactate 7%	+++	++	F07
NaCl 6% + Choline	++	+++	B08	Sodium lactate 8%	+++	+++	F08
NaCl 6% + Phosphorylcholine	+++	+++	B09	Sodium lactate 9%	+++	+++	F09
NaCl 6% + Creatine	++	+++	B10	Sodium lactate 10%	+++	+++	F10
NaCl 6% + Creatinine	+++	+++	B11	Sodium lactate 11%	+++	+++	F11
NaCl 6% + L-Carnitine	++	+++	B12	Sodium lactate 12%	+++	+++	F12
NaC1 6% + KCl	+++	+++	C01	Sodium phosphate pH 7 20 mM	++	+	G01
NaCl 6% + *L*-proline	+++	++	C02	Sodium phosphate pH 7 50 mM	++	−	G02
NaCl 6%+ *N*-Acethyl-*L*-glutamine	+++	+++	C03	Sodium phosphate pH 7 100 mM	+	−	G03
NaCl 6% + β-Glutamic acid	+++	+++	C04	Sodium phosphate pH 7 200 mM	+	−	G04
NaC1 6% + γ-Amino-*n*-butyric acid	+++	+++	C05	Sodium benzoate pH 5.2 20 mM	−	−	G05
NaC1 6% + Glutathione	+++	+++	C06	Sodium benzoate pH 5.2 50 mM	−	−	G06
NaCl 6% + Glycerol	++	+++	C07	Sodium benzoate pH 5.2 100 mM	−	−	G07
NaCl 6% + Trehalose	+++	+	C08	Sodium benzoate pH 5.2 200 mM	−	−	G08
NaCl 6% + Trimethylamine-*N*-oxide	+++	+++	C09	Ammonium sulfate pH 8 10 mM	+++	+++	G09
NaC1 6% + Trimethylamine	+++	+++	C10	Ammonium sulfate pH 8 20 mM	+++	+++	G10
NaC1 6% + Octopine	+++	+++	C11	Ammonium sulfate pH 8–50 mM	+++	+++	G11
NaC1 6% + Trigonelline	+++	+++	C12	Ammonium sulfate pH 8 100 mM	+++	+++	G12
Potassium chloride 3%	+++	+	D01	Sodium nitrite 10 mM	++	+	H01
Potassium chloride 4%	+++	+++	D02	Sodium nitrate 20 mM	+++	+++	H02
Potassium chloride 5%	+++	+++	D03	Sodium nitrate 40 mM	+++	+++	H03
Potassium chloride 6%	+++	+++	D04	Sodium nitrate 60 mM	+++	+	H04
Sodium sulfate 2%	+	+	D05	Sodium nitrate 80 mM	+++	+++	H05
Sodium sulfate 3%	++	+	D06	Sodium nitrate 100 mM	+++	+++	H06
Sodium sulfate 4%	++	+	D07	Sodium nitrite 10 mM	+++	+++	H07
Sodium sulfate 5%	++	+	D08	Sodium nitrite 20 mM	+++	+++	H08
Ethylene glycol 5%	++	−	D09	Sodium nitrite 40 mM	+	−	H09
Ethylene glycol 10%	+++	−	D10	Sodium nitrite 60 mM	+	−	H10
Ethylene glycol 15%	+++	+++	D11	Sodium nitrite 80 mM	+	−	H11
Ethylene glycol 20%	+++	−	D12	Sodium nitrite 100 mM	+	−	H12

Growth states: +++ represents good growth, ++ represents moderate growth, + represents weak growth. − represents no growth.

Sporulation: +++ represents sporulation is abundant, ++ represents sporulation is moderate, + represents sporulation is low, − represents no sporulation.

### Metabolic phenotypic analysis of *Piriformospora indica* under different pH values

A total of 96 pieces of information on pH metabolic phenotypes were obtained, and *P. indica* could grow normally in 88 pH environments (88/96 tested, plate PM10, Wells A01-H12; Table 6). The pH range for *P. indica* growth was 3.5–10, with the optimal pH for growth being approximately 5.0. In a pH 4.5 environment, *P. indica* was able to grow normally in all amino acids (PM10, B01-D12). In contrast, in a pH 9.5 environment, it could not grow normally in eight tested amino acids. Among the pH conditions where growth was normal, the conditions that detected spore production accounted for 53.41%. The amino acid decarboxylase and deaminase activities of microorganisms were tested under pH 4.5 and 9.5 conditions using the B1-D12 and E1-G12 wells of the PM10 plate, respectively. The results showed that *P. indica* has very strong amino acid decarboxylase activity (100% substrate utilization) and relatively strong deaminase activity (77.78% substrate utilization).

**Table 6:** Metabolic phenotypic characteristics of *Piriformospora indica* under different pH environments in PM10.

**pH and substrate**	**Growth**	**Sporulation**	**Well**	**pH and substrate**	**Growth**	**Sporulation**	**Well**
**PM 10**							
pH 3.5	+++	+++	A01	pH 9.5	+	−	E01
pH 4	+++	+++	A02	pH 9.5+ *L*-Alanine	+	−	E02
pH 4.5	++	++	A03	pH 9.5+ *L*-Arginine	+	−	E03
pH 5	+++	+	A04	pH 9.5+ *L*-Asparagine	+	−	E04
pH 5.5	+++	+	A05	pH 9.5+ *L*-Aspartic Acid	+	−	E05
pH 6	++	+	A06	pH 9.5+ *L*-Glutamic Acid	+	−	E06
pH 7	++	+	A07	pH 9.5+ *L*-Glutamine	++	−	E07
pH 8	++	−	A08	pH 9.5+ Glycine	+	−	E08
pH 8.5	+	−	A09	pH 9.5+ *L*-Histidine	+	−	E09
pH 9	+	−	A10	pH 9.5+ *L*-Isoleucine	+	−	E10
pH 9.5	+	−	A11	pH 9.5+ *L*-Leucine	+	−	E11
pH 10	+	−	A12	pH 9.5+ *L*-Lysine	+	−	E12
pH 4.5	+++	++	B01	pH 9.5+ *L*-Methionine	+	−	F01
pH 4.5+ *L*-Alanine	+++	+++	B02	pH 9.5+ *L*-Phenylalanine	+	−	F02
pH 4.5+ *L*-Arginine	+++	++	B03	pH 9.5+ *L*-Proline	**-**	−	F03
pH 4.5+ *L*-Asparagine	+++	++	B04	pH 9.5+ *L*-Serine	+	−	F04
pH 4.5+ *L*-Aspartic Acid	+++	+++	B05	pH 9.5+ *L*-Threonine	+	−	F05
pH 4.5+ *L*-Glutamic Acid	+++	+++	B06	pH 9.5+ *L*-Tryptophan	+	−	F06
pH 4.5+ *L*-Glutamine	+++	+++	B07	pH 9.5+ *L*-Tyrosine	−	−	F07
pH 4.5+ Glycine	+++	+++	B08	pH 9.5+ *L*-Valine	+	−	F08
pH 4.5+ *L*-Glutamic Acid	+++	+++	B09	pH 9.5+ Hydroxy-*L*-Proline	+	−	F09
pH 4.5+ *L*-Isoleucine	+++	+++	B10	pH 9.5+ *L*-Ornithine	+	−	F10
pH 4.5+ *L*-Leucine	+++	+++	B11	pH 9.5+ *L*-Homoarginine	−	−	F11
pH 4.5+ *L*-Lysine	+++	+++	B12	pH 9.5+ *L*-Homoserine	+	−	F12
pH 4.5+ *L*-Methionine	+++	+++	C01	pH 9.5+ Anthranilic acid	+	−	G01
pH 4.5+ *L*-Phenylalanine	+++	+++	C02	pH 9.5+ *L*-Norleucine	−	−	G02
pH 4.5+ *L*-Proline	+++	+++	C03	pH 9.5+ *L*-Norvaline	+	−	G03
pH 4.5+ *L*-Serine	+++	+++	C04	pH 9.5+ Agmatine	+	−	G04
pH 4.5+ *L*-Threonine	+++	+++	C05	pH 9.5+ Cadaverine	+	−	G05
pH 4.5+ *L*-Tryptophan	+++	+++	C06	pH 9.5+ Putrescine	+	−	G06
pH 4.5+ *L*-Tyrosine	+++	+++	C07	pH 9.5+ Histamine	−	−	G07
pH 4.5+ *L*-Valine	+++	+++	C08	pH 9.5+ Phenylethylamine	−	−	G08
pH 4.5+ Hydroxy-*L*-Proline	+++	+++	C09	pH 9.5+ Tyramine	−	++	G09
pH 4.5+ *L*-Ornithine	+++	++	C10	pH 9.5+ Creatine	−	−	G10
pH 4.5+ *L*-Homoarginine	+++	+++	C11	pH 9.5+ Trimethylamine-*N*-oxide	+	−	G11
pH 4.5+ *L*-Homoserine	+++	+++	C12	pH 9.5+ Urea	+	−	G12
pH 4.5+ Anthranilic acid	+	−	D01	*X*-Caprylate	++	−	H01
pH 4.5+ *L*-Norleucine	+++	+++	D02	*X-α-D*-Glucoside	+++	+	H02
pH 4.5+ *L*-Norvaline	+++	+++	D03	*X-β-D*-Glucoside	+++	+	H03
pH 4.5+ α- Amino-*N*-butyric acid	+++	++	D04	*X-α-D*-Galactoside	++	−	H04
pH 4.5+ *P*-Aminobenzoate	++	++	D05	*X-β-D*-Galactoside	++	−	H05
pH 4.5+ *L*-Cysteicacid	+++	++	D06	*X-α-D-*Glucuronide	++	+	H06
pH 4.5+ *D*-Lysine	+++	+++	D07	*X-β-D*-GIucuronide	++	−	H07
pH 4.5+ 5-Hydroxy Lysine	+++	+++	D08	*X-β-D*-Glucosaminide	++	−	H08
pH 4.5 +5-Hydroxy Tryptophan	+++	+++	D09	*X-β*-D-Galactosaminide	++	+	H09
pH 4.5+ *D, L*-Diaminopimelic acid	+	−	D10	*X-α-D-*Mannoside	++	−	H10
pH 4.5+ Trimethylamine-*N*-oxide	+++	+++	D11	*X*-PO_4_	+++	−	H11
pH 4.5+ Urea	+++	+++	D12	*X*-SO_4_	+++	+	H12

Growth states: +++ represents good growth, ++ represents moderate growth, + represents weak growth. − represents no growth.

Sporulation: +++ represents sporulation is abundant, ++ represents sporulation is moderate, + represents sporulation is low, − represents no sporulation.

## Discussion

*P. indica* possesses most of the functions of AM fungi for host plants, but the key difference is its ability to be cultured *in vitro*. In this study, we monitored the growth dynamics of *P. indica* on five common microbial culture media, including four fungal culture media and one bacterial culture medium. According to a regression equation that was derived to describe the relationship between colony diameter and growth days on different culture media, *P. indica* showed the fastest hyphal growth rate in the PDA culture medium. Previous studies have mainly used PDA medium and Kaefer medium (Kumar et al., 2011; Kashyap et al., 2023) for *P. indica* cultivation. However, the Kaefer medium, despite being considered optimal for growth, sporulation, and preservation of *P. indica*, is complex to prepare and expensive due to its requirement of more than 20 components. This poses challenges for its widespread application in laboratory or production settings (Attri and Varma, 2018; Kumar et al., 2023). Our study findings indicate that although PDA culture medium could induce spore production of *P. indica*, the sporulation time was long and the spore yield was low, making it difficult to meet the needs of strain preservation, metabolic phenotype testing, or other basic research. In contrast, this study demonstrates that the OA culture medium is the most suitable for sporulation, with the shortest sporulation time and the highest spore yield. The optimum medium for sporulation is coincident with some fungi, for instance, *Epicoccum latusicollum* (Li et al., 2023a) and *Valdensinia heterodoxa* (Zhao and Shamoun, 2006), but it is contrary to other fungi, including *Alternaria alternata* (Masangkay et al., 2000). OA culture medium, made from simple and inexpensive raw materials, contains a suitable carbon-to-nitrogen ratio and abundant vitamins and minerals. At the same time, the nutrients, especially nitrogen sources, in OA are not as abundant as those in PDA, YD, AEA, etc., making it favorable for the spore production of *P. indica*. The most suitable medium for *P. indica* can be used to provide the required biomass and spore quantity quickly for production or scientific research.

As a consequence, we employed an OA culture medium for subsequent Biolog PM metabolic phenotype experiments on *P. indica*. Compared with traditional microbial metabolism measurement methods, this high-throughput metabolic phenomics technology offers the advantages of high throughput, fast speed, and high accuracy (Khatri et al., 2013). It can dynamically reflect the biochemical processes of microbial cell metabolism in real-time (Kashyap et al., 2021). Biolog PM metabolic phenotype experiments have been used in the metabolic phenotype research of various microorganisms, including *Helicobacter pylori* (Lee et al., 2017), *A. alternate* (Wang H. C. et al., 2015), and *Phytophthora parasitica* (Wang M. S. et al., 2015). Our study obtained metabolic phenotype information from *P. indica* on various metabolic substrates and different environmental conditions, revealing a wide metabolic range of available carbon sources (87.89%), which is much higher than that of some plant pathogens, such as *Botrytis cinerea* (Wang et al., 2018)*, Rhizopus oryzae* (Li et al., 2023b), and *E. latusicollum* (Li et al., 2023a). The available carbon sources accounted for 17%, 54.21%, and 61.58% individually. This characteristic allows *P. indica* to be co-cultivated with plant seedlings, colonizing their roots during the seedling stage. At this stage, due to the limited types of carbon sources in plants, some pathogenic bacteria are difficult to grow normally. However, *P. indica* can utilize a wider range of carbon sources, which enhances its growth and spore production. After successful colonization, *P. indica* has great potential to enhance the plant's disease resistance. In the carbon sources of PM1-2, it is interesting that some of the carbon sources that cannot be metabolized by *P. indica* are isomers of metabolizable substrates, for example, α-Methyl-*D*-Galactoside cannot be utilized, while β-Methyl-*D*-Galactoside can be moderately utilized. However, isomers of some substrates, such as α-Methyl-*D*-Glucoside and β-Methyl-*D*-Glucoside, can be moderately utilized.

Furthermore, *P. indica* exhibits a relatively extensive range of metabolizable nitrogen sources (87.63%), including amino acids, dipeptides, or tripeptides, which is higher than that of some plant pathogens, such as *B. cinerea* (63% of available amino acid nitrogen substrates and 80% of available peptide nitrogen substrates) (Wang et al., 2018). Its metabolic ability on phosphorus sources (96.61%) and sulfur sources (100%) is significantly higher than that of certain plant pathogens. For instance, *Pseudomonas syringae* showed active metabolism with 10.17% phosphorus sources and metabolized none of the tested sulfur sources (Guo et al., 2017; Manzar et al., 2022a). It is worth noting that the sporulation rate of *P. indica* was 70.66% among all available carbon sources, while among all available nitrogen sources, only specific substances, such as Adenosine, *D, L*-Lactamide, Xanthosine, Ethanolamine, Ethylamine, and *N*-Butylamine induced spore production. This may be attributed to excessive nitrogen sources in the nitrogen source metabolism experiments, resulting in promoting mycelium growth but hindering sporulation. Therefore, it is necessary to select suitable types of nitrogen sources and control the appropriate carbon-to-nitrogen ratio to facilitate the production of *P. indica* spores in cultivation.

*P. indica* demonstrated a wide range of tested biosynthetic pathways, reflecting its ability to utilize various substrates to produce complex products or to metabolize complex substrates into simpler products. These biosynthetic pathways may play a crucial role in its life activities. For example, they are involved in the production of energy, such as the tricarboxylic acid cycle, and the synthesis of substances such as amino acids, nucleotides, and fatty acids. The diversity of biosynthetic pathways allows microbes to utilize substrates and energy more efficiently, thereby increasing resilience and competitiveness. The wide range of biosynthetic pathways in *P. indica* coincides with its powerful capability of substrate utilization. *P. indica* can grow in various tested osmotic environments, but it cannot grow under the conditions of 20–200 mM sodium benzoate (pH 5.2). The osmotic pressure adaptability of *P. indica* is stronger than that of some pathogens, such as *R. oryzae* could not survive in 3–6% sodium formate, 4–7% urea, and 20–200 mM sodium benzoate (pH 5.2). The extensive osmotic pressure adaptability of *P. indica* allows it to grow normally under various conditions of dryness and watering (Wang H. C. et al., 2015). *P. indica* had a wide range of pH tolerance, with the activity of decarboxylase being higher than that of deaminase. Decarboxylase of the microbe generates alkaline amines by the catabolism of amino acids, which help counteract the low pH condition (Maurer et al., 2005). Deaminase generates ketonic acid through the catabolism of amino acids, which helps counteract high pH. The deaminase activity is significantly higher than that of *A. alternata* (Wang H. C. et al., 2015). Additionally, *P. indica* could produce spores in most of the growable osmotic pressure and pH conditions. Obviously, *P. indica* has a wide range of environmental adaptability, which is consistent with its ability to colonize a variety of plant hosts (Qiang et al., 2012; Manzar et al., 2022b; Mahawer et al., 2023).

The growth dynamics and the Biolog PM metabolic phenotype information obtained in this study for *P. indica* can be used to optimize the culture conditions, spore formation, and germination conditions of artificial culture media for *P. indica*. These findings aid in understanding its colonization ability on various plants and enhance the host's resilience, enabling better cultivation and utilization of its colonization on various plants and maximizing its function in enhancing resistance to plant diseases and stress after colonization. Simultaneously, it provides a reference for the metabolic phenotype of other species within the genus *Piriformospora*.

## Conclusion

This study indicates that among the five commonly used microbial media tested for *P. indica*, PDA was found to be the most suitable medium for mycelium growth, while OA was the most suitable medium for spore production. Biolog PM1-10 was used to assess the metabolic phenotype of *P. indica*, revealing its ability to metabolize 87.89% of the tested carbon sources, 87.63% of the tested nitrogen sources, 96.61% of the tested phosphorus sources, and 100% of the tested sulfur sources, with 92 kinds of tested biosynthetic pathways. *P. indica* could grow under 92 kinds of test osmotic pressures and 88 kinds of test pH conditions, indicating a wide range of substrate metabolism in terms of carbon, nitrogen, phosphorus, and sulfur sources, as well as a wide range of environmental adaptability. This study provides a theoretical basis for screening more suitable growth and sporulation conditions for *P. indica*, elucidating its adaptive mechanisms in symbiosis with plants, understanding the physiological reasons behind enhancing the resistance of host plants, and establishing preferable application strategies.

## Data availability statement

The original contributions presented in the study are included in the article/Supplementary material, further inquiries can be directed to the corresponding authors.

## Author contributions

J-rH: Conceptualization, Funding acquisition, Software, Writing – original draft, Writing – review & editing. J-mL: Conceptualization, Writing – original draft. H-yW: Conceptualization, Writing – original draft, Software. M-lS: Data curation, Writing – original draft. C-yH: Conceptualization, Writing – original draft. H-cW: Writing – original draft, Methodology, Conceptualization.
