# Supplementary material for: Analysis of growth dynamics in five different media and metabolic phenotypic characteristics of Piriformospora indica
Source: Front Microbiol. 2024 Jan 8;14:1301743. doi: 10.3389/fmicb.2023.1301743 (PMC10800966; doi:10.3389/fmicb.2023.1301743)
Supplement: Supplementary file 1 [file Data_Sheet_1.PDF]

Phenotype MicroArrays™

PM1 MicroPlate™ Carbon Sources

A1 Negative Control	A2 L-Arabinose	A3 N-Acetyl-D-Glucosamine	A4 D-Saccharic Acid	A5 Succinic Acid	A6 D-Galactose	A7 L-Aspartic Acid	A8 L-Proline	A9 D-Alanine	A10 D-Trehalose	A11 D-Mannose	A12 Dulcitol
B1 D-Serine	B2 D-Sorbitol	B3 Glycerol	B4 L-Fucose	B5 D-Glucuronic Acid	B6 D-Gluconic Acid	B7 D,L-α-Glycerol-Phosphate	B8 D-Xylose	B9 L-Lactic Acid	B10 Formic Acid	B11 D-Mannitol	B12 L-Glutamic Acid
C1 D-Glucose-6-Phosphate	C2 D-Galactonic Acid-γ-Lactone	C3 D,L-Malic Acid	C4 D-Ribose	C5 Tween 20	C6 L-Rhamnose	C7 D-Fructose	C8 Acetic Acid	C9 α-D-Glucose	C10 Maltose	C11 D-Melibiose	C12 Thymidine
D-1 L-Asparagine	D2 D-Aspartic Acid	D3 D-Glucosaminic Acid	D4 1,2-Propanediol	D5 Tween 40	D6 α-Keto-Glutaric Acid	D7 α-Keto-Butyric Acid	D8 α-Methyl-D-Galactoside	D9 α-D-Lactose	D10 Lactulose	D11 Sucrose	D12 Uridine
E1 L-Glutamine	E2 M-Tartaric Acid	E3 D-Glucose-1-Phosphate	E4 D-Fructose-6-Phosphate	E5 Tween 80	E6 α-Hydroxy Glutaric Acid-γ-Lactone	E7 α-Hydroxy Butyric Acid	E8 β-Methyl-D-Glucoside	E9 Adonitol	E10 Maltotriose	E11 2-Deoxy Adenosine	E12 Adenosine
F1 Glycyl-L-Aspartic Acid	F2 Citric Acid	F3 M-Inositol	F4 D-Threonine	F5 Fumaric Acid	F6 Bromo Succinic Acid	F7 Propionic Acid	F8 Mucic Acid	F9 Glycolic Acid	F10 Glyoxylic Acid	F11 D-Cellobiose	F12 Inosine
G1 Glycyl-L-Glutamic Acid	G2 Tricarballylic Acid	G3 L-Serine	G4 L-Threonine	G5 L-Alanine	G6 L-Alanyl-Glycine	G7 Acetoacetic Acid	G8 N-Acetyl-β-D-Mannosamine	G9 Mono Methyl Succinate	G10 Methyl Pyruvate	G11 D-Malic Acid	G12 L-Malic Acid
H1 Glycyl-L-Proline	H2 p-Hydroxy Phenyl Acetic Acid	H3 m-Hydroxy Phenyl Acetic Acid	H4 Tyramine	H5 D-Psicose	H6 L-Lyxose	H7 Glucuronamide	H8 Pyruvic Acid	H9 L-Galactonic Acid-γ-Lactone	H10 D-Galacturonic Acid	H11 Phenylethyl-amine	H12 2-Aminoethanol

PM2A MicroPlate™ Carbon Sources

A1 Negative Control	A2 Chondroitin Sulfate C	A3 α-Cyclodextrin	A4 β-Cyclodextrin	A5 γ-Cyclodextrin	A6 Dextrin	A7 Gelatin	A8 Glycogen	A9 Inulin	A10 Laminarin	A11 Mannan	A12 Pectin
B1 N-Acetyl-D-Galactosamine	B2 N-Acetyl-Neuraminic Acid	B3 β-D-Allose	B4 Amygdalin	B5 D-Arabinose	B6 D-Arabitol	B7 L-Arabitol	B8 Arbutin	B9 2-Deoxy-D-Ribose	B10 I-Erythritol	B11 D-Fucose	B12 3-0-β-D-Galacto-pyranosyl-D-Arabinose
C1 Gentiobiose	C2 L-Glucose	C3 Lactitol	C4 D-Melezitose	C5 Maltitol	C6 α-Methyl-D-Glucoside	C7 β-Methyl-D-Galactoside	C8 3-Methyl Glucose	C9 β-Methyl-D-Glucuronic Acid	C10 α-Methyl-D-Mannoside	C11 β-Methyl-D-Xyloside	C12 Palatinose
D1 D-Raffinose	D2 Salicin	D3 Sedoheptulosan	D4 L-Sorbose	D5 Stachyose	D6 D-Tagatose	D7 Turanose	D8 Xylitol	D9 N-Acetyl-D-Glucosaminitol	D10 γ-Amino Butyric Acid	D11 δ-Amino Valeric Acid	D12 Butyric Acid
E1 Capric Acid	E2 Caproic Acid	E3 Citraconic Acid	E4 Citramalic Acid	E5 D-Glucosamine	E6 2-Hydroxy Benzoic Acid	E7 4-Hydroxy Benzoic Acid	E8 β-Hydroxy Butyric Acid	E9 γ-Hydroxy Butyric Acid	E10 α-Keto Valeric Acid	E11 Itaconic Acid	E12 5-Keto-D-Gluconic Acid
F1 D-Lactic Acid Methyl Ester	F2 Malonic Acid	F3 Melibionic Acid	F4 Oxalic Acid	F5 Oxalomalic Acid	F6 Quinic Acid	F7 D-Ribono-1,4-Lactone	F8 Sebacic Acid	F9 Sorbic Acid	F10 Succinamic Acid	F11 D-Tartaric Acid	F12 L-Tartaric Acid
G1 Acetamide	G2 L-Alaninamide	G3 N-Acetyl-L-Glutamic Acid	G4 L-Arginine	G5 Glycine	G6 L-Histidine	G7 L-Homoserine	G8 Hydroxy-L-Proline	G9 L-Isoleucine	G10 L-Leucine	G11 L-Lysine	G12 L-Methionine
H1 L-Ornithine	H2 L-Phenylalanine	H3 L-Pyroglutamic Acid	H4 L-Valine	H5 D,L-Carnitine	H6 Sec-Butylamine	H7 D.L-Octopamine	H8 Putrescine	H9 Dihydroxy Acetone	H10 2,3-Butanediol	H11 2,3-Butanone	H12 3-Hydroxy 2-Butanone

BIOLOG

Phenotype MicroArrays™

PM3B MicroPlate™ Nitrogen Sources

A1<br>Negative Control	A2<br>Ammonia	A3<br>Nitrite	A4<br>Nitrate	A5<br>Urea	A6<br>Biuret	A7<br>L-Alanine	A8<br>L-Arginine	A9<br>L-Asparagine	A10<br>L-Aspartic Acid	A11<br>L-Cysteine	A12<br>L-Glutamic Acid
B1<br>L-Glutamine	B2<br>Glycine	B3<br>L-Histidine	B4<br>L-Isoleucine	B5<br>L-Leucine	B6<br>L-Lysine	B7<br>L-Methionine	B8<br>L-Phenylalanine	B9<br>L-Proline	B10<br>L-Serine	B11<br>L-Threonine	B12<br>L-Tryptophan
C1<br>L-Tyrosine	C2<br>L-Valine	C3<br>D-Alanine	C4<br>D-Asparagine	C5<br>D-Aspartic Acid	C6<br>D-Glutamic Acid	C7<br>D-Lysine	C8<br>D-Serine	C9<br>D-Valine	C10<br>L-Citrulline	C11<br>L-Homoserine	C12<br>L-Ornithine
D-1<br>N-Acetyl-D,L-Glutamic Acid	D2<br>N-Phthaloyl-L-Glutamic Acid	D3<br>L-Pyroglutamic Acid	D4<br>Hydroxylamine	D5<br>Methylamine	D6<br>N-Amylamine	D7<br>N-Butylamine	D8<br>Ethylamine	D9<br>Ethanolamine	D10<br>Ethylenediamine	D11<br>Putrescine	D12<br>Agmatine
E1<br>Histamine	E2<br>β-Phenylethyl-amine	E3<br>Tyramine	E4<br>Acetamide	E5<br>Formamide	E6<br>Glucuronamide	E7<br>D,L-Lactamide	E8<br>D-Glucosamine	E9<br>D-Galactosamine	E10<br>D-Mannosamine	E11<br>N-Acetyl-D-Glucosamine	E12<br>N-Acetyl-D-Galactosamine
F1<br>N-Acetyl-D-Mannosamine	F2<br>Adenine	F3<br>Adenosine	F4<br>Cytidine	F5<br>Cytosine	F6<br>Guanine	F7<br>Guanosine	F8<br>Thymine	F9<br>Thymidine	F10<br>Uracil	F11<br>Uridine	F12<br>Inosine
G1<br>Xanthine	G2<br>Xanthosine	G3<br>Uric Acid	G4<br>Alloxan	G5<br>Allantoin	G6<br>Parabanic Acid	G7<br>D,L-α-Amino-N-Butyric Acid	G8<br>γ-Amino-N-Butyric Acid	G9<br>ε-Amino-N-Caproic Acid	G10<br>D,L-α-Amino-Caprylic Acid	G11<br>δ-Amino-N-Valeric Acid	G12<br>α-Amino-N-Valeric Acid
H1<br>Ala-Asp	H2<br>Ala-Gln	H3<br>Ala-Glu	H4<br>Ala-Gly	H5<br>Ala-His	H6<br>Ala-Leu	H7<br>Ala-Thr	H8<br>Gly-Asn	H9<br>Gly-Gln	H10<br>Gly-Glu	H11<br>Gly-Met	H12<br>Met-Ala

PM4A MicroPlate™ Phosphorus and Sulfur Sources

A1<br>Negative Control	A2<br>Phosphate	A3<br>Pyrophosphate	A4<br>Trimeta-phosphate	A5<br>Tripoly-phosphate	A6<br>Triethyl Phosphate	A7<br>Hypophosphite	A8<br>Adenosine- 2'-monophosphate	A9<br>Adenosine- 3'-monophosphate	A10<br>Adenosine- 5'-monophosphate	A11<br>Adenosine-2',3'-cyclic monophosphate	A12<br>Adenosine-3',5'-cyclic monophosphate
B1<br>Thiophosphate	B2<br>Dithiophosphate	B3<br>D,L-α-Glycerol Phosphate	B4<br>β-Glycerol Phosphate	B5<br>Carbamyl Phosphate	B6<br>D-2-Phospho-Glyceric Acid	B7<br>D-3-Phospho-Glyceric Acid	B8<br>Guanosine- 2'-monophosphate	B9<br>Guanosine- 3'-monophosphate	B10<br>Guanosine- 5'-monophosphate	B11<br>Guanosine-2',3'-cyclic monophosphate	B12<br>Guanosine-3',5'-cyclic monophosphate
C1<br>Phosphoenol Pyruvate	C2<br>Phospho-Glycolic Acid	C3<br>D-Glucose-1-Phosphate	C4<br>D-Glucose-6-Phosphate	C5<br>2-Deoxy-D-Glucose 6-Phosphate	C6<br>D-Glucosamine-6-Phosphate	C7<br>6-Phospho-Gluconic Acid	C8<br>Cytidine- 2'-monophosphate	C9<br>Cytidine- 3'-monophosphate	C10<br>Cytidine- 5'-monophosphate	C11<br>Cytidine- 2',3'-cyclic monophosphate	C12<br>Cytidine- 3',5'-cyclic monophosphate
D1<br>D-Mannose-1-Phosphate	D2<br>D-Mannose-6-Phosphate	D3<br>Cysteamine-S-Phosphate	D4<br>Phospho-L-Arginine	D5<br>O-Phospho-D-Serine	D6<br>O-Phospho-L-Serine	D7<br>O-Phospho-L-Threonine	D8<br>Uridine- 2'-monophosphate	D9<br>Uridine- 3'-monophosphate	D10<br>Uridine- 5'-monophosphate	D11<br>Uridine- 2',3'-cyclic monophosphate	D12<br>Uridine- 3',5'-cyclic monophosphate
E1<br>O-Phospho-D-Tyrosine	E2<br>O-Phospho-L-Tyrosine	E3<br>Phosphocreatine	E4<br>Phosphoryl Choline	E5<br>O-Phosphoryl-Ethanolamine	E6<br>Phosphono Acetic Acid	E7<br>2-Aminoethyl Phosphonic Acid	E8<br>Methylene Diphosphonic Acid	E9<br>Thymidine- 3'-monophosphate	E10<br>Thymidine- 5'-monophosphate	E11<br>Inositol Hexaphosphate	E12<br>Thymidine 3',5'- cyclic monophosphate
F1<br>Negative Control	F2<br>Sulfate	F3<br>Thiosulfate	F4<br>Tetrathionate	F5<br>Thiophosphate	F6<br>Dithiophosphate	F7<br>L-Cysteine	F8<br>D-Cysteine	F9<br>L-Cysteinyl-Glycine	F10<br>L-Cysteic Acid	F11<br>Cysteamine	F12<br>L-Cysteine Sulfinic Acid
G1<br>N-Acetyl-L-Cysteine	G2<br>S-Methyl-L-Cysteine	G3<br>Cystathionine	G4<br>Lanthionine	G5<br>Glutathione	G6<br>D,L-Ethionine	G7<br>L-Methionine	G8<br>D-Methionine	G9<br>Glycyl-L-Methionine	G10<br>N-Acetyl-D,L-Methionine	G11<br>L- Methionine Sulfoxide	G12<br>L-Methionine Sulfone
H1<br>L-Djenkolic Acid	H2<br>Thiourea	H3<br>1-Thio-β-D-Glucose	H4<br>D,L-Lipoamide	H5<br>Taurocholic Acid	H6<br>Taurine	H7<br>Hypotaurine	H8<br>p-Amino Benzene Sulfonic Acid	H9<br>Butane Sulfonic Acid	H10<br>2-Hydroxyethane Sulfonic Acid	H11<br>Methane Sulfonic Acid	H12<br>Tetramethylene Sulfone

Phenotype MicroArrays™

PM5 MicroPlate™ Nutrient Supplements

A1 Negative Control	A2 Positive Control	A3 L-Alanine	A4 L-Arginine	A5 L-Asparagine	A6 L-Aspartic Acid	A7 L-Cysteine	A8 L-Glutamic Acid	A9 Adenosine-3',5'-cyclic monophosphate	A10 Adenine	A11 Adenosine	A12 2'-Deoxy Adenosine
B1 L-Glutamine	B2 Glycine	B3 L-Histidine	B4 L-Isoleucine	B5 L-Leucine	B6 L-Lysine	B7 L-Methionine	B8 L-Phenylalanine	B9 Guanosine-3',5'-cyclic monophosphate	B10 Guanine	B11 Guanosine	B12 2'-Deoxy Guanosine
C1 L-Proline	C2 L-Serine	C3 L-Threonine	C4 L-Tryptophan	C5 L-Tyrosine	C6 L-Valine	C7 L-isoleucine + L-Valine	C8 trans-4-Hydroxy L-Proline	C9 (5) 4-Amino-Imidazole-4(5)-Carboxamide	C10 Hypoxanthine	C11 Inosine	C12 2'-Deoxy Inosine
D1 L-Ornithine	D2 L-Citrulline	D3 Chorismic Acid	D4 (-)Shikimic Acid	D5 L-Homoserine Lactone	D6 D-Alanine	D7 D-Aspartic Acid	D8 D-Glutamic Acid	D9 D,L-α,ε-Diamino-pimelic Acid	D10 Cytosine	D11 Cytidine	D12 2'-Deoxy Cytidine
E1 Putrescine	E2 Spermidine	E3 Spermine	E4 Pyridoxine	E5 Pyridoxal	E6 Pyridoxamine	E7 β-Alanine	E8 D-Pantothenic Acid	E9 Orotic Acid	E10 Uracil	E11 Uridine	E12 2'-Deoxy Uridine
F1 Quinolinic Acid	F2 Nicotinic Acid	F3 Nicotinamide	F4 β-Nicotinamide Adenine Dinucleotide	F5 δ-Amino-Levulinic Acid	F6 Hematin	F7 Deferoxamine Mesylate	F8 D-(+)-Glucose	F9 N-Acetyl D-Glucosamine	F10 Thymine	F11 Glutathione (reduced form)	F12 Thymidine
G1 Oxaloacetic Acid	G2 D-Biotin	G3 Cyano-Cobalamine	G4 p-Amino-Benzoic Acid	G5 Folic Acid	G6 Inosine + Thiamine	G7 Thiamine	G8 Thiamine Pyrophosphate	G9 Riboflavin	G10 Pyrrolo-Quinoline Quinone	G11 Menadione	G12 Myo-Inositol
H1 Butyric Acid	H2 D,L-α-Hydroxy-Butyric Acid	H3 α-Ketobutyric Acid	H4 Caprylic Acid	H5 D,L-α-Lipoic Acid (oxidized form)	H6 D,L-Mevalonic Acid	H7 D,L-Carnitine	H8 Choline	H9 Tween 20	H10 Tween 40	H11 Tween 60	H12 Tween 80

PM6 MicroPlate™ Peptide Nitrogen Sources

A1 Negative Control	A2 Positive Control: L-Glutamine	A3 Ala-Ala	A4 Ala-Arg	A5 Ala-Asn	A6 Ala-Glu	A7 Ala-Gly	A8 Ala-His	A9 Ala-Leu	A10 Ala-Lys	A11 Ala-Phe	A12 Ala-Pro
B1 Ala-Ser	B2 Ala-Thr	B3 Ala-Trp	B4 Ala-Tyr	B5 Arg-Ala	B6 Arg-Arg	B7 Arg-Asp	B8 Arg-Gln	B9 Arg-Glu	B10 Arg-Ile	B11 Arg-Leu	B12 Arg-Lys
C1 Arg-Met	C2 Arg-Phe	C3 Arg-Ser	C4 Arg-Trp	C5 Arg-Tyr	C6 Arg-Val	C7 Asn-Glu	C8 Asn-Val	C9 Asp-Asp	C10 Asp-Glu	C11 Asp-Leu	C12 Asp-Lys
D1 Asp-Phe	D2 Asp-Trp	D3 Asp-Val	D4 Cys-Gly	D5 Gln-Gln	D6 Gln-Gly	D7 Glu-Asp	D8 Glu-Glu	D9 Glu-Gly	D10 Glu-Ser	D11 Glu-Trp	D12 Glu-Tyr
E1 Glu-Val	E2 Gly-Ala	E3 Gly-Arg	E4 Gly-Cys	E5 Gly-Gly	E6 Gly-His	E7 Gly-Leu	E8 Gly-Lys	E9 Gly-Met	E10 Gly-Phe	E11 Gly-Pro	E12 Gly-Ser
F1 Gly-Thr	F2 Gly-Trp	F3 Gly-Tyr	F4 Gly-Val	F5 His-Asp	F6 His-Gly	F7 His-Leu	F8 His-Lys	F9 His-Met	F10 His-Pro	F11 His-Ser	F12 His-Trp
G1 His-Tyr	G2 His-Val	G3 Ile-Ala	G4 Ile-Arg	G5 Ile-Gln	G6 Ile-Gly	G7 Ile-His	G8 Ile-Ile	G9 Ile-Met	G10 Ile-Phe	G11 Ile-Pro	G12 Ile-Ser
H1 Ile-Trp	H2 Ile-Tyr	H3 Ile-Val	H4 Leu-Ala	H5 Leu-Arg	H6 Leu-Asp	H7 Leu-Glu	H8 Leu-Gly	H9 Leu-Ile	H10 Leu-Leu	H11 Leu-Met	H12 Leu-Phe

BIOLOG

Phenotype MicroArrays™

PM7 MicroPlate™ Peptide Nitrogen Sources

A1<br>Negative Control	A2<br>Positive Control: L-Glutamine	A3<br>Leu-Ser	A4<br>Leu-Trp	A5<br>Leu-Val	A6<br>Lys-Ala	A7<br>Lys-Arg	A8<br>Lys-Glu	A9<br>Lys-Ile	A10<br>Lys-Leu	A11<br>Lys-Lys	A12<br>Lys-Phe
B1<br>Lys-Pro	B2<br>Lys-Ser	B3<br>Lys-Thr	B4<br>Lys-Trp	B5<br>Lys-Tyr	B6<br>Lys-Val	B7<br>Met-Arg	B8<br>Met-Asp	B9<br>Met-Gln	B10<br>Met-Glu	B11<br>Met-Gly	B12<br>Met-His
C1<br>Met-Ile	C2<br>Met-Leu	C3<br>Met-Lys	C4<br>Met-Met	C5<br>Met-Phe	C6<br>Met-Pro	C7<br>Met-Trp	C8<br>Met-Val	C9<br>Phe-Ala	C10<br>Phe-Gly	C11<br>Phe-Ile	C12<br>Phe-Phe
D1<br>Phe-Pro	D2<br>Phe-Ser	D3<br>Phe-Trp	D4<br>Pro-Ala	D5<br>Pro-Asp	D6<br>Pro-Gln	D7<br>Pro-Gly	D8<br>Pro-Hyp	D9<br>Pro-Leu	D10<br>Pro-Phe	D11<br>Pro-Pro	D12<br>Pro-Tyr
E1<br>Ser-Ala	E2<br>Ser-Gly	E3<br>Ser-His	E4<br>Ser-Leu	E5<br>Ser-Met	E6<br>Ser-Phe	E7<br>Ser-Pro	E8<br>Ser-Ser	E9<br>Ser-Tyr	E10<br>Ser-Val	E11<br>Thr-Ala	E12<br>Thr-Arg
F1<br>Thr-Glu	F2<br>Thr-Gly	F3<br>Thr-Leu	F4<br>Thr-Met	F5<br>Thr-Pro	F6<br>Trp-Ala	F7<br>Trp-Arg	F8<br>Trp-Asp	F9<br>Trp-Glu	F10<br>Trp-Gly	F11<br>Trp-Leu	F12<br>Trp-Lys
G1<br>Trp-Phe	G2<br>Trp-Ser	G3<br>Trp-Trp	G4<br>Trp-Tyr	G5<br>Tyr-Ala	G6<br>Tyr-Gln	G7<br>Tyr-Glu	G8<br>Tyr-Gly	G9<br>Tyr-His	G10<br>Tyr-Leu	G11<br>Tyr-Lys	G12<br>Tyr-Phe
H1<br>Tyr-Trp	H2<br>Tyr-Tyr	H3<br>Val-Arg	H4<br>Val-Asn	H5<br>Val-Asp	H6<br>Val-Gly	H7<br>Val-His	H8<br>Val-Ile	H9<br>Val-Leu	H10<br>Val-Tyr	H11<br>Val-Val	H12<br>ϒ-Glu-Gly

PM8 MicroPlate™ Peptide Nitrogen Sources

A1<br>Negative Control	A2<br>Positive Control: L-Glutamine	A3<br>Ala-Asp	A4<br>Ala-Gln	A5<br>Ala-Ile	A6<br>Ala-Met	A7<br>Ala-Val	A8<br>Asp-Ala	A9<br>Asp-Gln	A10<br>Asp-Gly	A11<br>Glu-Ala	A12<br>Gly-Asn
B1<br>Gly-Asp	B2<br>Gly-Ile	B3<br>His-Ala	B4<br>His-Glu	B5<br>His-His	B6<br>Ile-Asn	B7<br>Ile-Leu	B8<br>Leu-Asn	B9<br>Leu-His	B10<br>Leu-Pro	B11<br>Leu-Tyr	B12<br>Lys-Asp
C1<br>Lys-Gly	C2<br>Lys-Met	C3<br>Met-Thr	C4<br>Met-Tyr	C5<br>Phe-Asp	C6<br>Phe-Glu	C7<br>Gln-Glu	C8<br>Phe-Met	C9<br>Phe-Tyr	C10<br>Phe-Val	C11<br>Pro-Arg	C12<br>Pro-Asn
D1<br>Pro-Glu	D2<br>Pro-Ile	D3<br>Pro-Lys	D4<br>Pro-Ser	D5<br>Pro-Trp	D6<br>Pro-Val	D7<br>Ser-Asn	D8<br>Ser-Asp	D9<br>Ser-Gln	D10<br>Ser-Glu	D11<br>Thr-Asp	D12<br>Thr-Gln
E1<br>Thr-Phe	E2<br>Thr-Ser	E3<br>Trp-Val	E4<br>Tyr-Ile	E5<br>Tyr-Val	E6<br>Val-Ala	E7<br>Val-Gln	E8<br>Val-Glu	E9<br>Val-Lys	E10<br>Val-Met	E11<br>Val-Phe	E12<br>Val-Pro
F1<br>Val-Ser	F2<br>β-Ala-Ala	F3<br>β-Ala-Gly	F4<br>β-Ala-His	F5<br>Met-β-Ala	F6<br>β-Ala-Phe	F7<br>D-Ala-D-Ala	F8<br>D-Ala-Gly	F9<br>D-Ala-Leu	F10<br>D-Leu-D-Leu	F11<br>D-Leu-Gly	F12<br>D-Leu-Tyr
G1<br>ϒ-Glu-Gly	G2<br>ϒ-D-Glu-Gly	G3<br>Gly-D-Ala	G4<br>Gly-D-Asp	G5<br>Gly-D-Ser	G6<br>Gly-D-Thr	G7<br>Gly-D-Val	G8<br>Leu-β-Ala	G9<br>Leu-D-Leu	G10<br>Phe-β-Ala	G11<br>Ala-Ala-Ala	G12<br>D-Ala-Gly-Gly
H1<br>Gly-Gly-Ala	H2<br>Gly-Gly-D-Leu	H3<br>Gly-Gly-Gly	H4<br>Gly-Gly-Ile	H5<br>Gly-Gly-Leu	H6<br>Gly-Gly-Phe	H7<br>Val-Tyr-Val	H8<br>Gly-Phe-Phe	H9<br>Leu-Gly-Gly	H10<br>Leu-Leu-Leu	H11<br>Phe-Gly-Gly	H12<br>Tyr-Gly-Gly

BIOLOG

Phenotype MicroArrays™

PM9 MicroPlate ™ Osmolytes

A1<br>NaCl 1%	A2<br>NaCl 2%	A3<br>NaCl 3%	A4<br>NaCl 4%	A5<br>NaCl 5%	A6<br>NaCl 5.5%	A7<br>NaCl 6%	A8<br>NaCl 6.5%	A9<br>NaCl 7%	A10<br>NaCl 8%	A11<br>NaCl 9%	A12<br>NaCl 10%
B1<br>NaCl 6%	B2<br>NaCl 6% +<br>Betaine	B3<br>NaCl 6% +<br>N-N Dimethyl<br>glycine	B4<br>NaCl 6% +<br>Sarcosine	B5<br>NaCl 6% +<br>Dimethyl<br>sulphonyl<br>propionate	B6<br>NaCl 6% +<br>MOPS	B7<br>NaCl 6% +<br>Ectoine	B8<br>NaCl 6% +<br>Choline	B9<br>NaCl 6% +<br>Phosphoryl<br>choline	B10<br>NaCl 6% +<br>Creatine	B11<br>NaCl 6% +<br>Creatinine	B12<br>NaCl 6% +<br>L- Carnitine
C1<br>NaCl 6% +<br>KCl	C2<br>NaCl 6% +<br>L-proline	C3<br>NaCl 6% +<br>N-Acethyl<br>L-glutamine	C4<br>NaC1 6% +<br>β-Glutamic acid	C5<br>NaC1 6% +<br>γ–Amino -n-<br>butyric acid	C6<br>NaC1 6% +<br>Glutathione	C7<br>NaCl 6% +<br>Glycerol	C8<br>NaC1 6% +<br>Trehalose	C9<br>NaC1 6% +<br>Trimethylamine<br>-N-oxide	C10<br>NaC1 6% +<br>Trimethylamine	C11<br>NaCl 6% +<br>Octopine	C12<br>NaC1 6% +<br>Trigonelline
D-1<br>Potassium<br>chloride<br>3%	D2<br>Potassium<br>chloride<br>4%	D3<br>Potassium<br>chloride<br>5%	D4<br>Potassium<br>chloride<br>6%	D5<br>Sodium sulfate<br>2%	D6<br>Sodium sulfate<br>3%	D7<br>Sodium sulfate<br>4%	D8<br>Sodium sulfate<br>5%	D9<br>Ethylene glycol<br>5%	D10<br>Ethylene glycol<br>10%	D11<br>Ethylene glycol<br>15%	D12<br>Ethylene glycol<br>20%
E1<br>Sodium formate<br>1%	E2<br>Sodium formate<br>2%	E3<br>Sodium formate<br>3%	E4<br>Sodium formate<br>4%	E5<br>Sodium formate<br>5%	E6<br>Sodium formate<br>6%	E7<br>Urea<br>2%	E8<br>Urea<br>3%	E9<br>Urea<br>4%	E10<br>Urea<br>5%	E11<br>Urea<br>6%	E12<br>Urea<br>7%
F1<br>Sodium Lactate<br>1%	F2<br>Sodium Lactate<br>2%	F3<br>Sodium Lactate<br>3%	F4<br>Sodium Lactate<br>4%	F5<br>Sodium Lactate<br>5%	F6<br>Sodium Lactate<br>6%	F7<br>Sodium Lactate<br>7%	F8<br>Sodium Lactate<br>8%	F9<br>Sodium Lactate<br>9%	F10<br>Sodium Lactate<br>10%	F11<br>Sodium Lactate<br>11%	F12<br>Sodium Lactate<br>12%
G1<br>Sodium<br>Phosphate pH 7<br>20mM	G2<br>Sodium<br>Phosphate pH 7<br>50mM	G3<br>Sodium<br>Phosphate pH 7<br>100mM	G4<br>Sodium<br>Phosphate pH 7<br>200mM	G5<br>Sodium<br>Benzoate pH<br>5.2<br>20mM	G6<br>Sodium<br>Benzoate pH<br>5.2<br>50mM	G7<br>Sodium<br>Benzoate pH5.2<br>100mM	G8<br>Sodium<br>Benzoate pH<br>5.2<br>200mM	G9<br>Ammonium<br>sulfate pH8<br>10mM	G10<br>Ammonium<br>sulfate pH 8<br>20mM	G11<br>Ammonium<br>sulfate pH 8<br>50mM	G12<br>Ammonium<br>sulfate pH8<br>100mM
H1<br>Sodium Nitrate<br>10mM	H2<br>Sodium Nitrate<br>20mM	H3<br>Sodium Nitrate<br>40mM	H4<br>Sodium Nitrate<br>60mM	H5<br>Sodium Nitrate<br>80mM	H6<br>Sodium Nitrate<br>100mM	H7<br>Sodium Nitrite<br>10mM	H8<br>Sodium Nitrite<br>20mM	H9<br>Sodium Nitrite<br>40mM	H10<br>Sodium Nitrite<br>60mM	H11<br>Sodium Nitrite<br>80mM	H12<br>Sodium Nitrite<br>100mM

PM10 MicroPlate ™ pH

A1<br>pH 3.5	A2<br>pH 4	A3<br>pH 4.5	A4<br>pH 5	A5<br>pH 5.5	A6<br>pH 6	A7<br>pH 7	A8<br>pH 8	A9<br>pH 8.5	A10<br>pH 9	A11<br>pH 9.5	A12<br>pH 10
B1<br>pH 4.5	B2<br>pH 4.5 +<br>L-Alanine	B3<br>pH 4.5 +<br>L-Arginine	B4<br>pH 4.5 +<br>L-Asparagine	B5<br>pH 4.5 +<br>L-Aspartic Acid	B6<br>pH 4.5 +<br>L-Glutamic<br>Acid	B7<br>pH 4.5 +<br>L-Glutamine	B8<br>pH 4.5 +<br>Glycine	B9<br>pH 4.5 +<br>L-Histidine	B10<br>pH 4.5 +<br>L-Isoleucine	B11<br>pH 4.5 +<br>L-Leucine	B12<br>pH 4.5 +<br>L-Lysine
C1<br>pH 4.5 +<br>L-Methionine	C2<br>pH 4.5 +<br>L-<br>Phenylalanine	C3<br>pH 4.5 +<br>L-Proline	C4<br>pH 4.5 +<br>L-Serine	C5<br>pH 4.5 +<br>L-Threonine	C6<br>pH 4.5 +<br>L-Tryptophan	C7<br>pH 4.5 +<br>L-Tyrosine	C8<br>pH 4.5 +<br>L-Valine	C9<br>pH 4.5 +<br>Hydroxy-<br>L-Proline	C10<br>pH 4.5 +<br>L-Ornithine	C11<br>pH 4.5 +<br>L-Homoarginine	C12<br>pH 4.5 +<br>L-Homoserine
D-1<br>pH 4.5 +<br>Anthranilic acid	D2<br>pH 4.5 +<br>L-Norleucine	D3<br>pH 4.5 +<br>L-Norvaline	D4<br>pH 4.5 +<br>α- Amino-N-<br>butyric acid	D5<br>pH 4.5 +<br>p-<br>Aminobenzoate	D6<br>pH 4.5 +<br>L-Cysteic acid	D7<br>pH 4.5 +<br>D-Lysine	D8<br>pH 4.5 +<br>5-Hydroxy<br>Lysine	D9<br>pH 4.5 +<br>5-Hydroxy<br>Tryptophan	D10<br>pH 4.5 +<br>D,L-Diamino<br>pimelic acid	D11<br>pH 4.5 +<br>Trimethyl<br>amine-N-oxide	D12<br>pH 4.5 +<br>Urea
E1<br>pH 9.5	E2<br>pH 9.5 +<br>L-Alanine	E3<br>pH 9.5 +<br>L-Arginine	E4<br>pH 9.5 +<br>L-Asparagine	E5<br>pH 9.5 +<br>L-Aspartic Acid	E6<br>pH 9.5 +<br>L-Glutamic<br>Acid	E7<br>pH 9.5 +<br>L-Glutamine	E8<br>pH 9.5 +<br>Glycine	E9<br>pH 9.5 +<br>L-Histidine	E10<br>pH 9.5 +<br>L-Isoleucine	E11<br>pH 9.5 +<br>L-Leucine	E12<br>pH 9.5 +<br>L-Lysine
F1<br>pH 9.5 +<br>L-Methionine	F2<br>pH 9.5 +<br>L-<br>Phenylalanine	F3<br>pH 9.5 +<br>L-Proline	F4<br>pH 9.5 +<br>L-Serine	F5<br>pH 9.5 +<br>L-Threonine	F6<br>pH 9.5 +<br>L-Tryptophan	F7<br>pH 9.5 +<br>L-Tyrosine	F8<br>pH 9.5 +<br>L-Valine	F9<br>pH 9.5 +<br>Hydroxy-<br>L-Proline	F10<br>pH 9.5 +<br>L-Ornithine	F11<br>pH 9.5 +<br>L-Homoarginine	F12<br>pH 9.5 +<br>L-Homoserine
G1<br>pH 9.5 +<br>Anthranilic acid	G2<br>pH 9.5 +<br>L-Norleucine	G3<br>pH 9.5 +<br>L-Norvaline	G4<br>pH 9.5 +<br>Agmatine	G5<br>pH 9.5 +<br>Cadaverine	G6<br>pH 9.5 +<br>Putrescine	G7<br>pH 9.5 +<br>Histamine	G8<br>pH 9.5 +<br>Phenylethylamin<br>e	G9<br>pH 9.5 +<br>Tyramine	G10<br>pH 9.5 +<br>Creatine	G11<br>pH 9.5 +<br>Trimethyl<br>amine-N-oxide	G12<br>pH 9.5 +<br>Urea
H1<br>X-Caprylate	H2<br>X–α-D-<br>Glucoside	H3<br>X-β-D-<br>Glucoside	H4<br>X-α-D-<br>Galactoside	H5<br>X-β-D-<br>Galactoside	H6<br>X-α- D-<br>Glucuronide	H7<br>X-β- D-<br>Glucuronide	H8<br>X-β-D-<br>Glucosaminide	H9<br>X-β-D-<br>Galactosaminid<br>e	H10<br>X-α-D-<br>Mannoside	H11<br>X-PO4	H12<br>X-SO4
